# Tumor microenvironmental cytokines bound to cancer exosomes determine uptake by cytokine receptor-expressing cells and biodistribution

**DOI:** 10.1038/s41467-021-23946-8

**Published:** 2021-06-10

**Authors:** Luize G. Lima, Sunyoung Ham, Hyunku Shin, Edna P. Z. Chai, Erica S. H. Lek, Richard J. Lobb, Alexandra F. Müller, Suresh Mathivanan, Belinda Yeo, Yeonho Choi, Belinda S. Parker, Andreas Möller

**Affiliations:** 1 Tumour Microenvironment Laboratory, QIMR Berghofer Medical Research Institute, Herston, QLD Australia; 2 School of Biomedical Sciences, Faculty of Health, Queensland University of Technology, Brisbane, QLD Australia; 3 Department of Bio-convergence Engineering, Korea University, Seoul, South Korea; 4 Faculty of Medicine, University of Queensland, Brisbane, QLD Australia; 5 School of Chemistry and Molecular Biosciences, Faculty of Science, University of Queensland, Brisbane, QLD Australia; 6 Department of Biochemistry and Genetics, La Trobe Institute for Molecular Science, La Trobe University, Melbourne, VIC Australia; 7 Olivia Newton-John Cancer Research Institute, Austin Hospital, Heidelberg, Melbourne, VIC Australia; 8 School of Biomedical Engineering, Korea University, Seoul, South Korea; 9 Department of Bioengineering, Korea University, Seoul, South Korea; 10 Cancer Immunology Program, Peter MacCallum Cancer Centre, Melbourne, VIC Australia; 11 Sir Peter MacCallum Department of Oncology, University of Melbourne, Parkville, VIC Australia; 12 Present Address: Centre for Personalized Nanomedicine, Australian Institute for Bioengineering and Nanotechnology (AIBN), University of Queensland, Brisbane, QLD Australia

**Keywords:** Breast cancer, Metastasis, Cytokines

## Abstract

Metastatic spread of a cancer to secondary sites is a coordinated, non-random process. Cancer cell-secreted vesicles, especially exosomes, have recently been implicated in the guidance of metastatic dissemination, with specific surface composition determining some aspects of organ-specific localization. Nevertheless, whether the tumor microenvironment influences exosome biodistribution has yet to be investigated. Here, we show that microenvironmental cytokines, particularly CCL2, decorate cancer exosomes via binding to surface glycosaminoglycan side chains of proteoglycans, causing exosome accumulation in specific cell subsets and organs. Exosome retention results in changes in the immune landscape within these organs, coupled with a higher metastatic burden. Strikingly, CCL2-decorated exosomes are directed to a subset of cells that express the CCL2 receptor CCR2, demonstrating that exosome-bound cytokines are a crucial determinant of exosome-cell interactions. In addition to the finding that cytokine-conjugated exosomes are detected in the blood of cancer patients, we discovered that healthy subjects derived exosomes are also associated with cytokines. Although displaying a different profile from exosomes isolated from cancer patients, it further indicates that specific combinations of cytokines bound to exosomes could likewise affect other physiological and disease settings.

## Abstract (web summary)

Cancer derived exosomes are reported to promote metastatic dissemination. Here the authors show that cytokines in the tumor microenvironment bind to exosomes via glycosaminoglycan side chains of proteoglycans, and these exosomes are preferentially taken up by specific cell lineages and organs to promote metastasis.

## Introduction

Metastasis is the process through which cancer cells spread to distal sites and is responsible for ~90% of cancer mortality^1^. It involves a highly coordinated cascade of biological events, regulated by inherent cancer cell-autonomous pathways, as well as dynamic interaction of cancer cells with various tumor-associated stromal lineages, including immune cells^2^. Metastasis-initiating tumor cells are reported to display essential intrinsic features, such as cellular plasticity, enhanced migratory and invasive capacities, resistance to apoptosis, and immune editing^2^. Additionally, primary tumors can release cytokines, growth factors and other protein factors capable of priming distant tissues for the arrival of circulating cancer cells, thereby creating a pre-metastatic niche capable of supporting metastatic outgrowth^3–5^.

Cancer cell-secreted vesicles, especially exosomes, are known as essential mediators of pre-metastatic niche formation^6–8^. Exosomes are small particles of endocytic origin with a size of 30–150 nm^9^. Secretion of exosomes by tumor cells significantly contributes to intercellular communication and subsequent reprogramming of the tumor microenvironment^8^. In addition to exosomal core proteins (such as CD9, CD63, Flotillin-1, HSP70, and TSG101), exosomes contain a variety of other proteins, nucleic acids, and lipids depending on the cell-of-origin^10–12^. Cancer cell-autonomous mechanisms of metastasis organotropism involve not only direct receptor/ligand interactions between the tumor cells and the distant organ microenvironment^13^, but also the preparation of pre-metastatic niches by cancer-derived exosomes in an organ-specific manner, which is partially determined by the integrin composition of the exosomes^14^. Yet, if and how the tumor microenvironment modulates cancer-derived exosomes systemic biodistribution, thereby influencing their organ-specific localization and thus function, has yet to be understood.

Here we show, using syngeneic mouse models and cancer patient samples, that tumor microenvironmental cytokines bind to cancer-derived exosomes via glycosaminoglycan (GAG) side chains of proteoglycans. These cytokine-bound exosomes are selectively taken up by cytokine-receptor-positive cells in specific tissues, resulting in changes in the immune landscape of these secondary organs, as well as in exosome biodistribution, with a consequent increase in metastasis.

## Results

### Tumor microenvironmental cytokines affect organ-specific exosome accumulation and promote metastasis

**Fig. 1:**
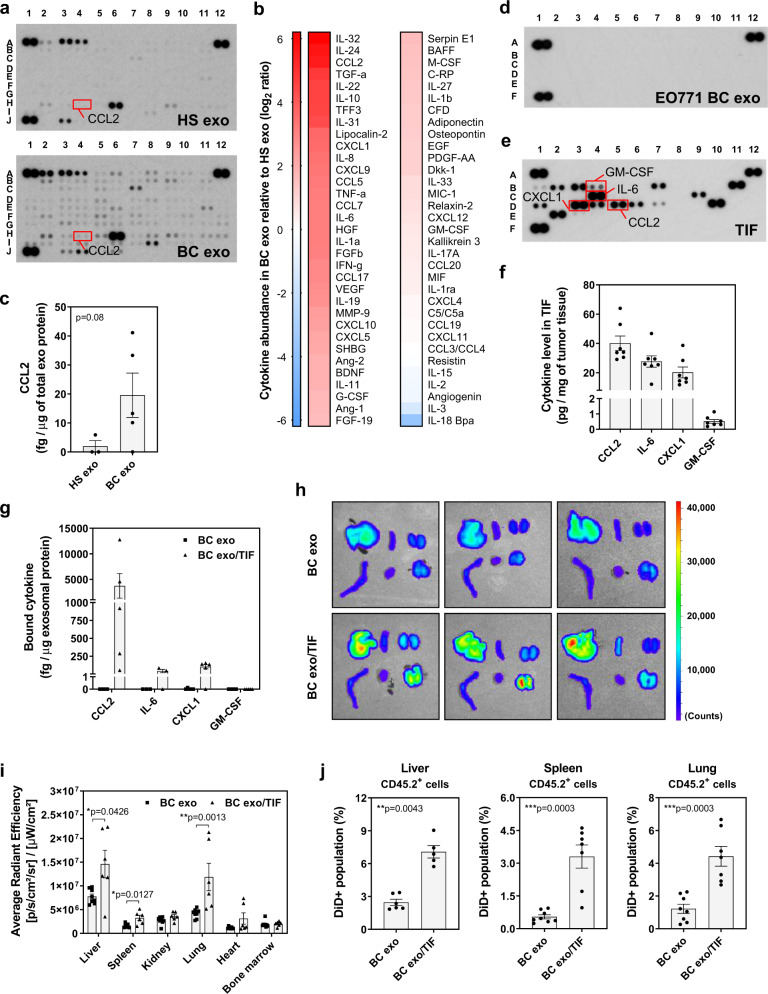
Binding of tumor microenvironmental cytokines affects BC exosomes biodistribution. **a** Cytokine arrays incubated with exosomes purified from plasma of healthy subject (HS exo) or breast cancer patients (BC exo). The respective CCL2 spot is highlighted (red box). **b** Heatmap representation of quantification of cytokine profile of plasma exosomes of BC patients (BC exo) relative to healthy subjects (HS exo) (*n* = 2 biologically independent samples/group). **c** Quantification of CCL2 in exosomes purified from plasma of healthy subjects (HS exo; *n* = 3 biologically independent samples) or BC patients (*n* = 5 biologically independent samples) by ELISA. **d**, **e** Cytokine arrays incubated with (**d**) cell culture-derived EO771 exosomes or (**e**) exosome-depleted TIF obtained from overnight cultures of minced EO771 tumors grown in C57Bl/6 mice. Representative images are shown. **f** Absolute amounts of CCL2, IL-6, CXCL1, and GM-CSF in exosome-depleted TIF as assessed by CBA (*n* = 7 biologically independent TIF preparations). The respective spots of each cytokine are highlighted in (**e**) (red boxes). **g** Binding of TIF-derived cytokines to cell culture-derived EO771 exosomes (BC exo/TIF; black triangles) as assessed by CBA. Exosomes alone served as controls (BC exo; black squares) (*n* = 5 independent experiments, except for IL-6 (*n* = 3 independent experiments)). **h**–**j** DiD-labeled cell culture-derived EO771 exosomes previously incubated (BC exo/TIF) or not (BC exo) with TIF were injected into C57Bl/6 wild-type (WT) mice, and biodistribution assessed 24 h after injection. **h** Representative ex vivo images of DiD fluorescence in various organs (clockwise from top-left: liver, spleen, kidney, lung, heart and bone marrow). **i** Quantification of DiD fluorescence in the same organs as shown in (**h**) (BC exo, *n* = 8 animals; black squares) (BC exo/TIF, *n* = 6 animals; black triangles). **j** Frequency of DiD^+^ population within CD45.2^+^ cells from liver (BC exo and BC exo/TIF, *n* = 6 and *n* = 5 animals, respectively), spleen (BC exo, and BC exo/TIF *n* = 8 animals and *n* = 7 animals, respectively) and lung (BC exo and BC exo/TIF, *n* = 8 and *n* = 7 animals, respectively) as assessed by flow cytometry. Data are presented as mean ± SEM. *p < 0.05, **p < 0.01, and ***p < 0.001 as analyzed by two-tailed Mann–Whitney *U*-test.

**Fig. 2:**
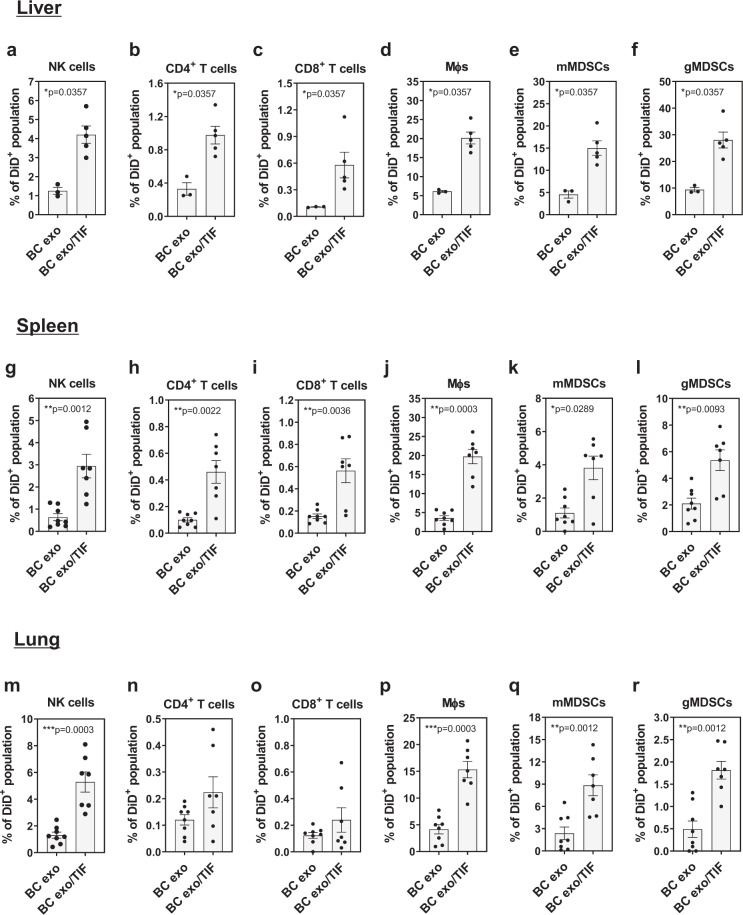
Increased uptake of TIF-conjugated exosomes by immune cell subsets in the liver (a-f), spleen (g-l) and lung (m-r). C57Bl/6 WT mice were injected with DiD-labeled cell culture-derived EO771 exosomes previously incubated (BC exo/TIF, *n* = 7 animals) or not (BC exo, *n* = 8 animals) with TIF. **a**–**r** Frequency of DiD^+^ population was assessed within distinct CD45^+^ immune cell subsets using flow cytometry: (**a**, **g**, **m**) NK cells (CD3^−^NK1.1^+^); (**b**, **h**, **n**) CD4^+^ T cells (CD3^+^CD4^+^); (**c**, **i**, **o**) CD8^+^ T cells (CD3^+^CD8^+^); (**d**, **j**, **p**) macrophages (Mɸ; CD11b^+^F4/80^+^); (**e**, **k**, **q**) mMDSCs (CD11b^+^Gr1^lo^); and (**f**, **l**, **r**) gMDSCs (CD11b^+^/Gr1^hi^). Data are presented as mean ± SEM. *p < 0.05, **p < 0.01, and ***p < 0.001 as analyzed by two-tailed Mann–Whitney *U*-test.

**Fig. 3:**
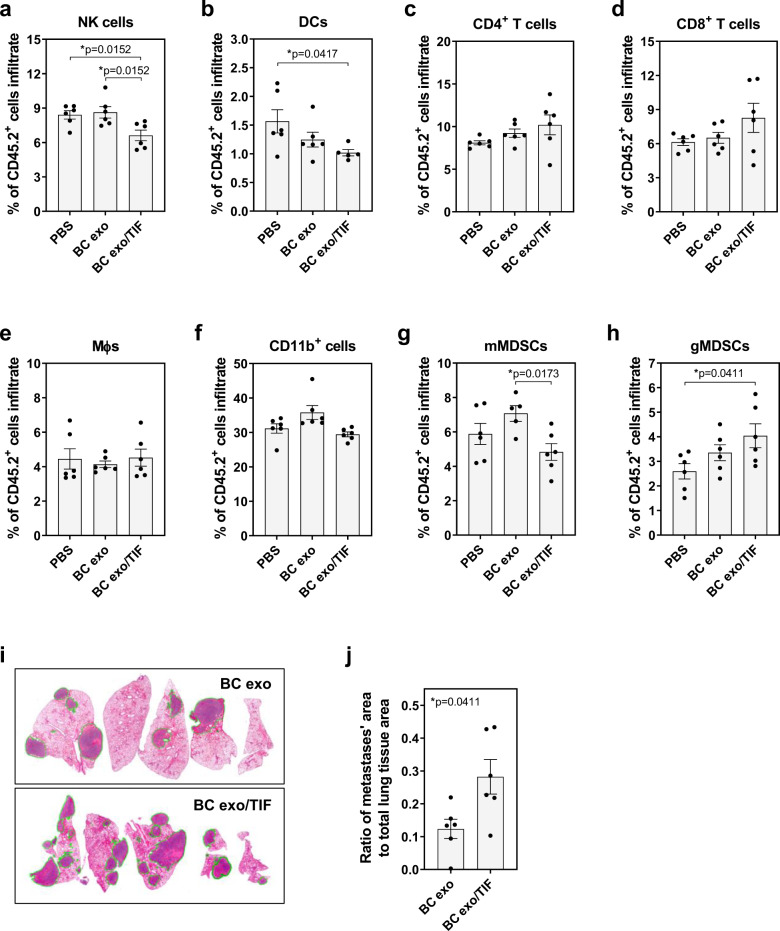
Cytokine-bound exosomes create a cancer-permissive environment to enhance metastasis. C57Bl/6 WT mice were injected with cell culture-derived EO771 exosomes, previously incubated (BC exo/TIF) or not (BC exo) with TIF, every 48 h, three times. PBS only-injected mice served as negative controls (*n* = 6 animals/group). **a**–**h** Frequency of distinct CD45.2^+^ immune cell subsets in the lung: **a** NK cells (CD3^−^NK1.1^+^), **b** dendritic cells (DCs; CD11c^+^MHCII^+^), **c** CD4^+^ T cells (CD3^+^CD4^+^), **d** CD8^+^ T cells (CD3^+^CD8^+^), **e** macrophages (Mɸ; CD11b^+^F4/80^+^), **f** myeloid cells (CD11b^+^), **g** mMDSCs (CD11b^+^Ly6C^hi^Ly6G^−^), and **h** gMDSCs (CD11b^+^/Ly6C^lo^/Ly6G^+^) as assessed by flow cytometry. **i**, **j** Mice pre-conditioned with cell culture-derived EO771 exosomes previously incubated (BC exo/TIF) or not (BC exo) with TIF received a single i.v. injection of EO771 cells, and metastatic burden in the lung was assessed (*n* = 6 animals/group). **i** Representative lung sections of each group (H&E staining; metastatic area outlined in green). **j** Ratio of metastases’ area to total lung tissue area (each data point represents a different mouse; one section was examined per mouse; all five lobes were analyzed and combined to determine metastases’ and total lung tissue areas per section). Data are presented as mean ± SEM. *p < 0.05, as analyzed by two-tailed Mann–Whitney *U*-test.

Analyzing exosomes isolated from plasma of healthy subjects and breast cancer (BC) patients (Supplementary Fig. 1 and Supplementary Table 1), we identified a number of cytokines and growth factors co-isolating with exosomes, with a greater abundance and variety found on BC patient-derived exosomes (Fig. 1a, b). In particular, higher levels of CCL2 and IL-6 were confirmed to be increased in exosome samples from BC patients when assessed by ELISA (Fig. 1c and Supplementary Fig. 2). In stark contrast, pure exosomes isolated from EO771 BC cells cultured in vitro were largely devoid of cytokines and growth factors (Fig. 1d). The cytokine composition of the tumor microenvironment depends on its cellular composition, as well as the cellular responses to the various cell-to-cell interactions, and is inherently complex. To evaluate the behavior of exosomes in such a cytokine milieu, we obtained exosome-depleted tumor interstitial fluid (TIF) from syngeneic, orthotopic EO771 cancer masses, which showed a range of cytokines and growth factors (Fig. 1e, f), including CCL2 and IL-6. Addition of EO771 cell culture-derived exosomes to this exosome-depleted TIF, with subsequent re-isolation of exosomes to remove free unbound soluble factors, showed an enrichment of specific exosome/cytokine associations (Fig. 1g). CCL2, IL-6 and CXCL1, for example, were readily co-isolated, whereas GM-CSF was not detectable (Fig. 1g). Evaluating the behavior of EO771 exosomes conjugated to TIF-derived cytokines in mice, we found that TIF-conjugated exosomes were retained in various organs at significantly higher abundance than control exosomes (Fig. 1h, i and Supplementary Fig. 3) and resulted in increased leukocyte uptake (Fig. 1j; see Supplementary Fig. 4a for gating strategy). Several immune cell lineages, including NK cells (CD3^−^NK1.1^+^), macrophages (CD11b^+^F4/80^+^) and monocytic and granulocytic myeloid-derived suppressor cells (mMDSCs, CD11b^+^Gr1^lo^; gMDSCs, CD11b^+^Gr1^hi^) in the liver, spleen, and lung had higher exosome uptake of TIF-exosomes than non-conjugated exosomes (Fig. 2; see Supplementary Fig. 4a, b for gating strategy). Interestingly, repeated injections of TIF-exosomes altered the immune cell composition of lungs (Fig. 3a–h), spleen (Supplementary Fig. 5), and liver (Supplementary Fig. 6) (see Supplementary Fig. 4b–e for gating strategy). These alterations include: a reduction of NK cells in both lungs (Fig. 3a) and spleen (Supplementary Fig. 5a); an increase of gMDSCs in the lungs (Fig. 3h); a reduced frequency of dendritic cells (DCs) in the liver (Supplementary Fig. 6b); and a higher frequency of Ly6C^−^ macrophages, which were reported to be associated with immunosuppressive responses^15,16^, in both spleen (Supplementary Fig. 5i) and liver (Supplementary Fig. 6i).

Pre-metastatic niches are thought to act as permissive environments for circulating cancer cells to adhere and grow^17^, so we induced pre-metastatic niche formation by repeated injections of exosomes previously incubated with TIF, followed by injection of syngeneic cancer cells. Conditioning of mice with TIF-conjugated exosomes significantly (*p* < 0.05) increased metastasis formation in the lungs compared to non-conjugated exosomes (Fig. 3i, j). Altogether, these data show that cytokines in the tumor microenvironment are capable of associating with cancer-derived exosomes, thereby inducing changes in the immune landscape of distal organs, and subsequent increase of metastatic burden.

### Cytokines bind to the external surface of cancer-derived exosomes via GAG side chains of proteoglycans

**Fig. 4:**
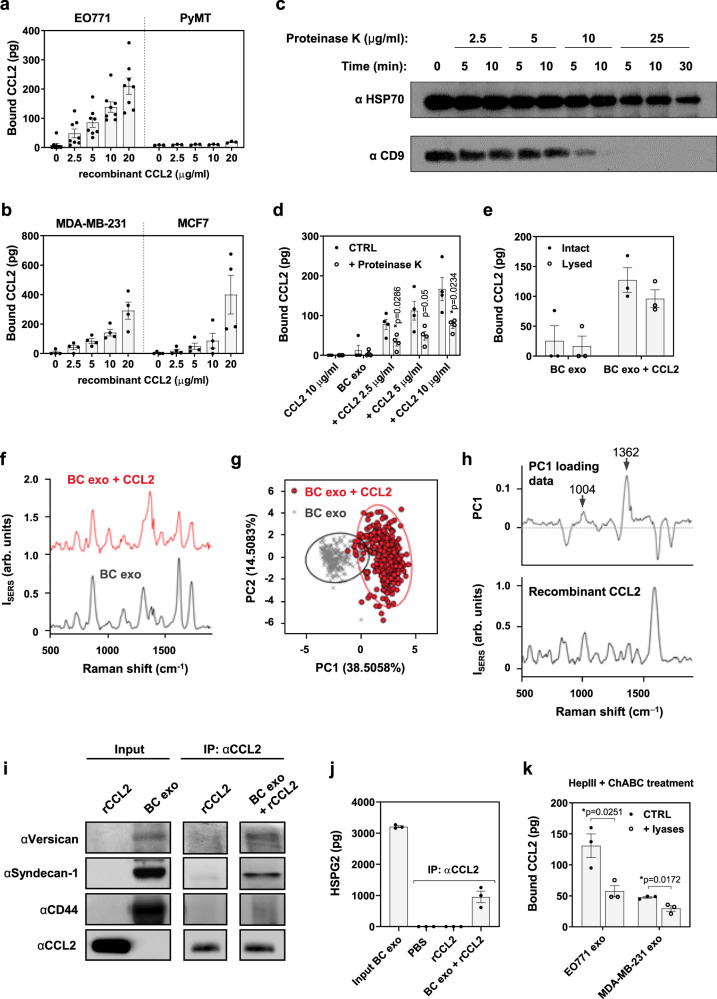
Exosomal GAG side chains of proteoglycans are responsible for cytokine binding to BC exosomes. **a**, **b** Concentration-dependent binding of CCL2 to **a** murine (EO771 and PyMT, *n* = 7 and *n* = 3 independent experiments, respectively) and **b** human (MDA-MB-231 and MCF7, *n* = 4 independent experiments) BC cell culture-derived exosomes. **c** EO771 exosomes were treated with increasing concentrations of Proteinase K, which was inactivated at different time points. Expression of intravesicular (HSP70) or surface (CD9) exosome markers was then assessed. Non-treated exosomes (first lane) served as control. Similar results were obtained from 3 independent experiments. Source data are provided as a Source Data file. **d**, **e** CCL2-conjugated EO771 exosomes (CTRL; black circles) were (**d**) treated with Proteinase K (*n* = 4 independent experiments; open circles) or (**e**) sonicated before CCL2 detection (*n* = 3 independent experiments; open circles). **f** SERS signal of exosome surface conjugated (BC exo + CCL2; red line) or not (BC exo; gray line) to CCL2. **g** PCA-based classification to identify the difference between the SERS spectra. The ellipses indicate 95% confidence ellipse for the plotted data (BC exo; gray diagonal crosses) (BC exo + CCL2; red circles). **h** The PC1 loading data that represent a dominant spectral feature of CCL2-conjugated exosomes (top) and the characteristic Raman spectrum of CCL2 alone (bottom). **i**, **j** Co-immunoprecipitation analysis of CCL2 and proteoglycans versican, syndecan-1 and CD44 (**i**) (similar results were obtained from three independent experiments), as well as HSPG2 (**j**) (*n* = 3 independent experiments), from MDA-MB-231 exosomes. Source data are provided as a Source Data file. **k** Binding of CCL2 to EO771 and MDA-MB-231 exosomes (CTRL; black circles) previously treated with HepIII and ChABC lyases (open circles) (*n* = 3 independent experiments). Data are presented as mean ± SEM. *p < 0.05 as analyzed by two-tailed Mann–Whitney *U*-test.

To evaluate the mechanism behind the association of cytokines with cancer-derived exosomes, we evaluated whether cytokines are localized within exosomes or bound to the exosome outer membrane. To investigate their sub-exosomal localization, we added recombinant human or mouse CCL2 and IL-6, both cytokines present in both plasma exosome isolates and TIF (Fig. 1 and Supplementary Fig. 2), to two human and two murine cancer cell line-derived exosomes. After incubation, we removed unbound cytokines by re-purifying exosomes. We indeed observed a concentration-dependent increase of both CCL2 (Fig. 4a, b) and IL-6 (Supplementary Fig. 7a, b) in exosome isolates in these settings. Of note, binding of CCL2 to murine PyMT cell-derived exosomes was comparatively lower than in all other settings (Fig. 4a). We further treated CCL2-bound BC exosomes with proteinase K at a concentration that led to the degradation of outer membrane proteins such as CD9, but not intravesicular HSP70 (Fig. 4c), confirming disruption was limited to the exosome surface. Treatment of the surface of BC exosomes with proteinase K (Fig. 4d, gray bars) indicated the majority of CCL2 is located on the exosome outer membrane. Also, no significant difference in CCL2 content was observed between lysed and intact vesicles (Fig. 4e), suggesting only minimal contribution of intravesicular cytokines to total exosomal CCL2 levels in this setting. We further corroborated these findings by analyzing the surface of exosomes after CCL2 incubation by surface-enhanced Raman spectroscopy (SERS), which detects molecular fingerprints from membrane proteins on exosomes^18–20^. Compared to exosomes without cytokine addition, BC exosomes pre-conjugated to CCL2 showed different spectral intensities at several bands (Fig. 4f). Principal Component Analysis (PCA) was then applied to identify major spectral patterns of the CCL2-conjugated exosomes. Exosomes conjugated with CCL2 could be clearly distinguished from non-conjugated ones, with their spectra mainly plotted on the positive side of principal component 1 (PC1) (Fig. 4g). SERS detects Raman spectra only in very close vicinity to a noble metal substrate, indicating most detectable CCL2 signals were derived from the vesicle surface. Furthermore, by comparing the PC1 loading data to the characteristic Raman signal of recombinant CCL2 alone, we observed that the positive peaks in PC1 that contributed to the CCL2-exosome distribution exhibited similar patterns to the CCL2 signal, especially around 1004 and 1362 cm^−1^ (Fig. 4h, see arrows). These observations indicate the difference between exosome samples was indeed induced by the SERS signal from CCL2.

Proteomic assessment of BC exosomes did not identify cognate receptors for most of the cytokines we detected associated with plasma exosomes (Supplementary Table 2)^11,12^. However, analyzing our mass spectrometry data^11^, we found an abundance of both heparan sulfate (HS) and chondroitin sulfate (CS) proteoglycans, including CD44 (HS/CS-proteoglycan), HSPG2 (HS-proteoglycan), glypican-1 (GPC-1; HS-proteoglycan), versican (VCAN; CS-proteoglycan) and syndecan-1 (SDC1; HS/CS-proteoglycan), in BC exosomes (Supplementary Fig. 7C^11^). Intriguinly, the proteoglycan profiles of the two murine BC cell-derived EO771 and PyMT exosomes are very different (Supplementary Fig. 7c). Quantitatively, analysis by ELISA showed MDA-MB-231 cell-derived exosomes exhibit a proteoglycan profile similar to that of EO771 (Supplementary Fig. 7d). GAGs present in proteoglycans have been shown to bind cytokines in different cellular contexts^21,22^. We therefore evaluated whether such a binding is also possible in an exosomal context and show that versican, syndecan-1 and CD44 (Fig. 4i), as well as HSPG2 (Fig. 4j), co-precipitate with exosomal-conjugated CCL2. Moreover, addition of heparinase III (HepIII) and chondroitinase ABC (ChABC) lyases, which catalyze degradation of HS and CS chains of proteoglycans, respectively, reduced CCL2 binding to exosomes (Fig. 4k). Overall, these data show that extracellular cytokines can bind to exosomes secreted by cancer cells via exosomal surface GAG side chains of proteoglycans.

### Cytokine binding alters exosome biodistribution and cell lineage-specific uptake

**Fig. 5:**
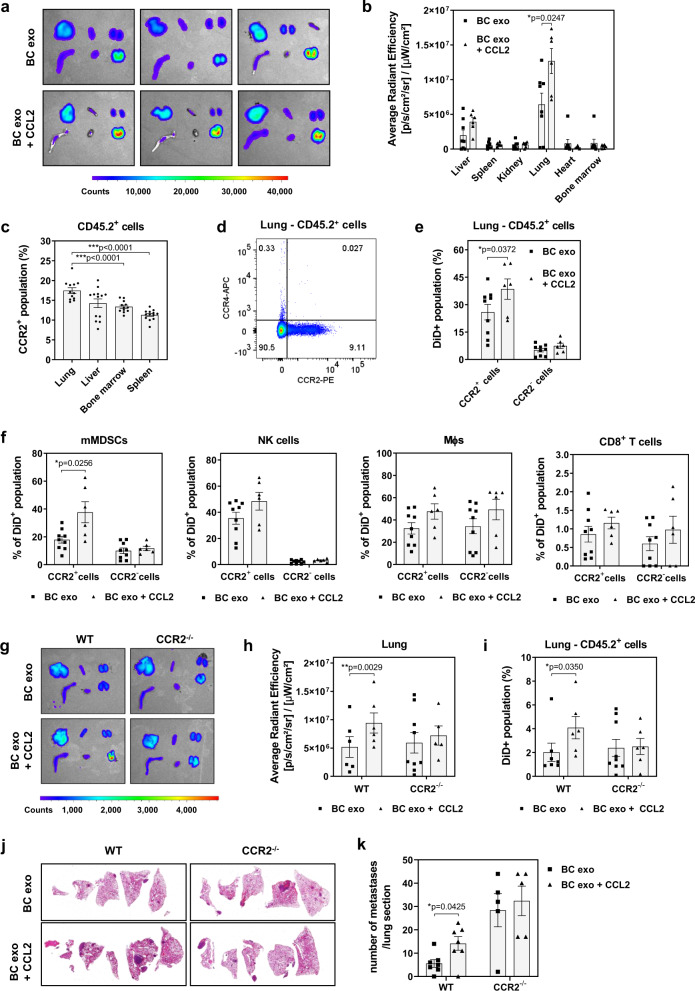
CCL2 binding affects the in vivo biodistribution of BC exosomes and facilitates BC metastasis. **a**, **b** DiD-labeled EO771 exosomes previously incubated (BC exo + CCL2, *n* = 6 animals) or not (BC exo, *n* = 8 animals) with CCL2 were injected into C57Bl/6 WT mice, and biodistribution assessed 24 h after injection. **a** Representative ex vivo images of DiD fluorescence in various organs from WT mice (clockwise from top-left: liver, spleen, kidney, lung, heart and bone marrow). **b** Quantification of DiD fluorescence in the same organs as shown in **a** (BC exo; black squares) (BC exo + CCL2; black triangles). **c** Frequency of CCR2^+^ population within lung (*n* = 12 animals), liver (*n* = 13 animals), bone marrow (*n* = 12 animals) and spleen (*n* = 13 animals) CD45.2^+^ cells as assessed by flow cytometry. **d** Frequency of CCR4^+^ and/or CCR2^+^ populations within CD45.2^+^ cells in the lung. **e**, **f** C57Bl/6 WT mice were injected with DiD-labeled EO771 exosomes previously incubated (BC exo + CCL2, *n* = 6 animals; black squares,) or not (BC exo, *n* = 9 animals; black triangles) with CCL2. **e** Frequency of DiD^+^ population within CD45.2^+^ cells in the lung. CCR2^+^ and CCR2^−^ subpopulations were gated and analyzed separately. **f** Frequency of DiD^+^ population within distinct CD45.2^+^ immune cell subsets. CCR2^+^ and CCR2^−^ subpopulations were gated and analyzed separately. **g**–**i** DiD-labeled EO771 exosomes previously incubated (BC exo + CCL2, *n* = 6 and *n* = 5 animals in the WT and CCR2^−/−^ groups, respectively; black triangles) or not (BC exo, *n* = 6 and *n* = 9 animals in the WT and CCR2^−/−^ groups, respectively; black squares) with CCL2 were injected into C57Bl/6 WT and CCR2^−/−^ mice, and biodistribution assessed 24 h after injection. **g** Representative ex vivo images of DiD fluorescence in various organs from WT and CCR2^−/−^ mice. **h** Quantification of DiD fluorescence in the lung as shown in **g**. **i** Frequency of DiD^+^ population within CD45.2^+^ cells in the lung of WT and CCR2^−/−^ mice. **j**, **k** Mice pre-conditioned with cell culture-derived EO771 exosomes previously incubated (BC exo + CCL2, *n* = 7 and *n* = 5 animals in the WT and CCR2^−/−^ groups, respectively; black triangles) or not (BC exo, *n* = 7 and *n* = 5 animals in the WT and CCR2^−/−^ groups, respectively; black squares) with CCL2 received a single i.v. injection of EO771 cells, and metastatic burden in the lung was assessed. **j** Representative lung sections of each group (H&E staining). **k** Number of metastatic foci per tissue section (each data point represents a different mouse; one section was examined per mouse; all five lobes were analyzed and combined to determine total number of metastases per section). Data are presented as mean ± SEM. *p < 0.05, **p < 0.01, and ***p < 0.001 as analyzed by two-tailed Mann–Whitney *U*-test.

To determine the mechanism underlying the altered TIF-conjugated exosome distribution observed above, CCL2-conjugated, fluorescently labeled EO771 BC cell-derived exosomes were intravenously injected into syngeneic mice. Ex vivo imaging of various organs (including liver, spleen, kidney, lung, heart, and bone marrow; Fig. 5a, b) demonstrated a significant increase in CCL2-conjugated exosome accumulation in the lungs (Fig. 5b), which was confirmed by fluorescence microscopy of lung tissue sections (Supplementary Fig. 8a). CCL2 preferentially binds to its receptor CCR2 and lung leukocytes are frequently CCR2^+^ (Fig. 5c). Conversely, CCR4, also reported to function as a receptor for CCL2, could not be detected in lung leukocytes (Fig. 5d). CCL2-conjugated exosomes were more efficiently taken up by lung CD45.2^+^CCR2^+^ leukocytes compared to non-conjugated exosomes, while CD45.2^+^CCR2^−^ leukocytes displayed no differential uptake (Fig. 5e). Especially in mMDSCs, which show distinct CCR2^+^ and CCR2^−^ populations (Supplementary Fig. 8b), we found CCL2-conjugated exosomes to be much more abundant in CCR2^+^ cells compared to CCR2^−^ cells, whereas unconjugated exosome uptake was similar (Fig. 5f). A trend to an increase in CCL2-conjugated exosomes uptake specifically by CCR2^+^ NK cells was also observed, although not statistically significant (Fig. 5f). In contrast to the altered bio- and cell lineage-distribution in WT mice (Fig. 5b, e), CCL2-conjuged BC exosomes exerted no effect on organ distribution (Fig. 5g), lung accumulation (Fig. 5h) or lung CD45.2^+^ cell uptake (Fig. 5i) in CCR2^−/−^ mice. Finally, conditioning of WT mice with CCL2-conjugated exosomes, followed by injection of syngeneic cancer cells, led to a significant (*p* < 0.05) increase in lung metastasis formation compared to non-conjugated exosomes, while no impact on BC metastasis was noticed in the lungs of CCR2^−/−^ mice (Fig. 5j and k). Interestingly, repeated injections of recombinant CCL2 alone did not affect BC metastasis when compared to a control group injected only with PBS (Supplementary Fig. 8c). More intringuinly, exosomes previously incubated with TIF did not affect the metastatic outgrowth of BC cells in the lungs of CCR2^−/−^ mice (Supplementary Fig. 8d), indicating a crutial role of exosome-bound CCL2 in BC metastatic dissemination.

**Fig. 6:**
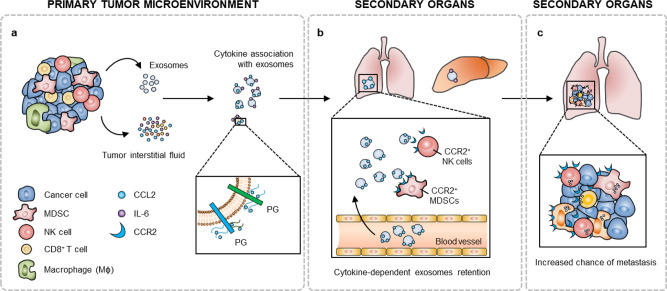
Summary of findings. **a** BC-derived exosomes are decorated by cytokines present in the tumor microenvironment. CCL2 (and likely other cytokines) bind to exosomal GAG side chains of proteoglycans (PG) such as CD44, HSPG2, syndecan-1 and versican. **b** After cytokine conjugation, BC exosomes are predominantly retained into organs such as lung, and display a high affinity and preferential uptake by CCR2^+^ immune cells, such as MDSCs and NK cells. These immune cells, which have interacted with cytokine-conjugated exosomes, contribute to the formation of a metastasis-favorable environment, promoting **c** subsequent metastatic progression.

Altogether, these data show that CCL2 binding to BC exosomes alters their systemic biodistribution as well as cell lineage-specific vesicle uptake, and likely contribute to the pro-metastatic changes induced in distal organs by TIF-conjugated BC exosomes (Fig. 3 and Supplementary Figs. 5 and 6), thereby facilitating BC metastasis (Fig. 6).

## Discussion

Exosomes and other extracellular vesicles (EV)s have been co-isolated with cytokines^23–25^. However, whether cytokines are present in the inner space of these vesicles or attached to their surface remains to be clarified. A comprehensive study analyzing different biological systems showed that EV-associated cytokines isolated from tissue explants and plasma are more often bound to the EV-surface^25^. In contrast, EVs produced by cultured T cells and monocytes predominantly carry cytokines in an encapsulated form, which can be modulated upon cell activation^25^. Similarly, we observed that exosomes obtained from human blood samples display a larger variety of cytokines when compared to cell culture-derived exosomes. These data indicate that in vivo EVs not only carry intrinsic molecules from their cell-of-origin, but are also decorated by cytokines and other soluble factors present within the tumor extracellular space. Importantly, since EVs produced by cell types other than tumor cells, including stromal and hematopoietic lineages, can also be present in the circulation, the contribution of non-tumor-derived EVs to the total pool of cytokines detected in our exosome isolates cannot be disregarded.

From a functional perspective, the localization of cytokines on exosomes and other EVs is likely to contribute to its biological activity. Since cytokine receptors are usually present on the cell surface, cytokines bound to exosomes may direct these small vesicles to target cells in a ligand/receptor dependent manner. Furthermore, this cytokine-exosome association may specifically modulate recipient cells that express a particular range of cytokine receptors, not only through the vesicle’s content uptake but also through cytokine/receptor signaling.

While cytokine receptors are largely absent in exosomes^11,12^, it appears that the proteoglycan composition provides a certain level of cytokine-binding selectivity. Exosomal proteoglycan profiles vary between different cancer cells^11,12^, supporting our finding that the same cytokine (i.e., CCL2) showed variations in its binding to exosomes from the different cancer cell lines tested, whereas different cytokines from the tumor interstitial fluid exhibited distinct levels of affinity to the same exosome. Moreover, some proteoglycans, such as Glypican-1, were found to be exclusively present in cancer-derived exosomes when compared to non-transformed cell- or healthy donor plasma-derived exosomes^26^. Different cancer types were shown to express distinct proteoglycan profiles correlated to cancer cell behavior and differentiation status^27^, which could be reflected in cancer exosomes.

In a cellular and extracellular matrix context, binding to proteoglycans has been previously reported to be essential for the in vivo functionality of cytokines, such as CCL2^22^. Furthermore, interactions between cytokines and GAGs side chains of proteoglycans were shown to be highly selective^28^. For example, CCL5 displayed the highest affinity for heparin among four different cytokines, while MIP-1a exhibited negligible binding to the same GAG^28^. On the other hand, all the cytokines tested exhibited selectivity in their association with distinct GAGs, such as HS and CS, although at varied levels, with CCL5 being the most selective one^28^. The finding that the integrin composition of cancer-derived exosomes determines the systemic accumulation of these vesicles^14^ neatly integrates with our results, suggesting cytokines and GAG side chains of proteoglycans may be also involved in the specific, exosome-intrinsic mechanisms of organotropism.

Besides the integrin profile of exosomes^14^, it has been long known that the expression of cytokine receptors on tumor cells influence their accumulation in selective secondary organs^13^. For example, the expression of CXCR4 on breast cancer cells has been reported to guide metastatic dissemination towards distal tissues that display abundance of the CXCR4 ligand CXCL12 (SDF-1)^13^. By demonstrating that the cytokine composition of the primary tumor microenvironment influences the biodistribution of tumor-derived exosomes, our work adds a cancer cell-independent control of metastatic guidance to the cancer cell-autonomous mechanisms described thus far^13,14^.

The composition of the tumor microenvironment has been shown to affect cancer cell survival in early stages of tumor growth, thereby determining the success of anti-cancer therapies, such as checkpoint inhibitor treatments^29^. However, most exosome and EV-based biomarker approaches currently focus on the abundance of these vesicles as an indicator. The work presented here, together with earlier studies^14^, suggests that simultaneous evaluation of the integrin, proteoglycan and cytokine features of exosomes will more comprehensively provide information on putative metastatic sites and determine successful therapeutic strategies.

## Methods

### Plasma collection from healthy donors and breast cancer patients

Plasma samples were prepared from blood^30^ collected from healthy female, age-matched subjects, or breast cancer patients (Supplementary Table S1). Briefly, blood was obtained from each subject in EDTA-coated tubes and allowed to sit at room temperature for 30 min. Whole blood was then centrifuged at 1,200 × *g* for 10 min at 4 °C to separate plasma. Plasma was transferred to a clean tube and centrifuged again at 1,800 × *g* for 10 min at 4 °C before being aliquoted, snap frozen on dry ice and stored at −80 °C until use. Ethical clearance for the use of the plasma samples was granted by the QIMR Berghofer Medical Research Institute Human Research Ethics Committee (P1499). Informed consent has been obtained from all participants.

### Mice

Female C57Bl/6 wild-type mice were used at 8-10 weeks of age and purchased from the Walter and Eliza Hall Institute (Melbourne, Australia). C57Bl/6 CCR2^−/−^ mice were bred and maintained at the QIMR Berghofer Medical Research Institute. All animal procedures were conducted in accordance with Australian National Health and Medical Research regulations on the use and care of experimental animals, and approved by the QIMR Berghofer Medical Research Institute Animal Ethics Committee (A12617M, P1499).

### Antibodies

The following primary and secondary antibodies were used for western blotting: mouse anti-flotillin-1 (1:1,000 dilution; BD Biosciences, cat. #610821), mouse anti-HSP70 (1:1,000 dilution; BD Biosciences, cat. #610608), goat anti-TSG101 (1:1,000 dilution; Santa Cruz Biotechnology, cat. #6037), rabbit anti-CD9 (1:3,000 dilution; abcam, cat. #92726), rabbit anti-GM130 (1:1,000 dilution; abcam, cat. #52649), rabbit anti-calnexin (1:,1000 dilution; Cell Signalling Technology, cat. #2679S), rabbit anti-syndecan-1 (1:1,000 dilution; abcam, cat. #128936), rabbit anti-versican (1:1,000 dilution; abcam, cat. #177480), mouse anti-CCL2 (1:1,000 dilution; BioLegend, cat. #502602), rat anti-CD44 (1:500 dilution; eBioscience, cat. #11-0441-81), goat anti-mouse HRP-conjugated (1:30,000 dilution; Pierce, cat. #1858413), goat anti-rabbit HRP-conjugated (1:10,000 dilution; Pierce, cat. #1858415), donkey anti-goat HRP-conjugated (1:250,000 dilution; Sigma-Aldrich, cat. #A5420), and goat anti-rat HRP-conjugated (1:2,000 dilution; R&D Systems, cat. #HAF005). The following fluorochrome-conjugated antibodies were used for flow cytometry: anti-CCR2 PE (1:200 dilution; R&D Systems, cat. #FAB5538P), anti-CCR4 APC (1:100 dilution; BioLegend, cat. #131211), anti-CD45.2 BV786 (1:300 dilution; BD Biosciences, cat. #563686), anti-CD3e FITC (1:300 dilution; eBioscience, cat. #11-003181), anti-NK1.1 e450 (1:300 dilution; eBioscience, cat. #48-5941-80), anti-CD8a PE (1:300 dilution; BD Biosciences, cat. #553033), anti-CD8a PE-Cy7 (1:300 dilution; eBioscience, cat. #25-0081-81), anti-CD4 BV510 (1:400 dilution; BD Biosciences, cat. #563106), anti-CD11c BV650 (1:200 dilution; BD Biosciences, cat. #564079), anti-Gr1 e450 (1:300 dilution; eBioscience, cat. #48-5931-80), anti-CD11b FITC (1:400 dilution; eBioscience, cat. #11-0112-81), anti-CD11b PE-Cy7 (1:300 dilution; eBioscience, cat. #25-0112-81), anti-F4/80 APC-e780 (1:300 dilution; eBioscience, cat. #47-4801-80), anti-Ly6C FITC (1:300 dilution; BD Biosciences, cat. #553104), anti-Ly6G PE (1:300 dilution; BD Biosciences, cat. #220461), and anti-MHCII BUV395 (1:300 dilution; BD Biosciences, cat. #743876).

### Cell culture

Murine and human breast cancer cell lines EO771^31^, PyMT^32^, MDA-MB-231 and MCF-7 (both obtained from ATCC) were maintained in Dulbecco’s modified Eagle medium (DMEM; Gibco™, cat. # 11995-073) supplemented with 5% fetal bovine serum (FBS; Gibco™, cat. # 10099-141) and 100 U/ml penicillin-streptomycin (Gibco™, cat. #15140-122), and grown in an incubator at 37 °C, 5% CO_2_. All cell lines were routinely tested negative for mycoplasma contamination. Authentication of human cell lines was verified using in-house STR profiling.

### EV-depleted medium preparation

Briefly, EV-depleted FBS-containing DMEM was prepared by overnight centrifugation (100,000 x *g*, 4 °C) of DMEM supplemented with 100 U/ml penicillin-streptomycin plus 20% (v/v) FBS. Supernatant was collected and filtered (0.22 µm). EV-depleted medium was then diluted with DMEM supplemented with penicillin-streptomycin only, in order to reach the final FBS concentration.

### Isolation of exosomes

Cells were seeded into 150 × 25 mm dishes at a concentration of 1 × 10^6^ cells/dish for EO771 and PyMT cell lines, and 1.5 × 10^6^ cells/dish for MDA-MB-231 and MCF-7 cell lines. After overnight adhesion, cells were washed with Dulbecco’s phosphate buffered saline (PBS; Gibco™, cat. #14190-144) and conditioned in EV-depleted 5% FBS-containing DMEM (15 ml/dish). Conditioned media were collected from 48-h cell cultures (cell viability > 95%), followed by centrifugation (500 x *g*; 10 min) and filtration (0.22 µm) to remove dead cells and large debris. Exosomes were pelleted by ultracentrifugation at 100,000 x *g* for 90 min at 4 °C and washed once in PBS. Further purification of exosomes was performed by overlaying exosome suspensions on qEV size exclusion chromatography columns (Izon Science Ltd) followed by sample concentration in Amicon Ultra-4 10-kDa nominal molecular weight centrifugal filter units (Merck Millipore) to a final volume of 200 µl for further analysis. For human plasma samples, 1 ml of processed plasma was directly overlaid onto qEV size exclusion columns (Izon Science Ltd) followed by sample concentration to a final volume of 100 µl.

### Transmission electron microscopy (TEM)

TEM imaging was performed using a JEOL 1011 transmission electron microscope at 60 kV^30^. Briefly, purified exosomes were fixed with paraformaldehyde and transferred to Formvar-carbon-coated electron microscopy grids. Grids were transferred to 1% (v/v) glutaraldehyde for 5 min, followed by eight washes with water. For contrast, grids were negatively stained with 1% (w/v) uranyl-oxalate solution, pH 7 for 5 min before transferring to methyl-cellulose-UA for 10 min. Excess fluid was removed and exosomes were finally imaged.

### Tunable resistive pulse sensing (TRPS)

Particle abundance and size distribution were assessed using the qNano system (Izon Science Ltd) by TRPS technology with NP100 nanopores and 100-nm calibration beads (CPC100)^30^.

### Western blotting

Exosome preparations and cell lysates were solubilized with either Laemmli sample buffer or RIPA buffer, respectively. Protein content was quantified using a standard Bradford assay or BCA assay, and analyzed by western blotting^30^. The membranes were probed with aforementioned antibodies. Full length images with molecular weight markers of all western blotting results shown in the manuscript are included in the Source Data file.

### Cytokine arrays

Cytokines in exosome preparations and TIF were detected using Proteome Profiler array kits (R&D Systems, human: cat. #ARY022B/mouse: cat. #ARY006), according to the manufacturers’ instructions. Forty micrograms of total protein were used. Arbitrary values of cytokine abundance were calculated as integrated densities of each dot plot normalized by the reference spots. Integrated densities were measured using the ImageJ software (v1.51w).

### TIF preparation

EO771 cells (5 × 10^5^/mouse) were injected into the 4th right mammary fat pad of C57Bl/6 mice. After 14 days, tumors were dissected, weighed, washed in PBS, and taken into 6-well plates. Tissues were minced in DMEM, and incubated overnight at 37 °C, 5% CO_2_. Conditioned media were collected and centrifuged at 500 x *g* for 10 min. The supernatants were filtered through 0.22 µm filters (Merck Millipore) and centrifuged at 100,000 x *g* for 90 min at 4^ o^C to deplete exosomes. Final supernatants were kept at −80 °C until use. TIF cytokine levels were assessed by BD™ Mouse Soluble Protein CBA Flex Set assay. Flow-cytometric acquisition was completed using a LSRFortessa™ (BD Biosciences) and the BD FACSDiva™ software (BD Biosciences, v8), and analysis was performed using the FCAP Array™ software (BD Biosciences, v3).

### Exosome conjugation to TIF

Exosomes (500 µg/ml) were incubated with 1 ml of TIF for 2 h at 37 °C under agitation. Exosomes were then purified using qEV columns (Izon Science Ltd) and cytokine binding assessed by BD™ Mouse Soluble Protein CBA Flex Set assay.

### DiD labeling of exosomes

Exosomes were fluorescently labeled using Vybrant® DiD (Life Technologies, cat. #V22887) according to manufacturer’s instructions with modifications^7^.

### In vivo tracking of fluorescently labeled exosomes

DiD-labeled exosomes were injected intravenously into syngeneic wild-type or CCR2^−/−^ C57Bl/6 mice (7.5  × 10^11^ particles/mouse). At 24 h after injection, tissues were harvested for ex vivo imaging. The intensity of fluorescence was quantified using the IVIS Spectrum and Living Image Software (PerkinElmer, v4.4) to assess tissue distribution of DiD-labeled exosomes. The average radiant efficiency of PBS-injected controls was subtracted from the average radiant efficiency of exosome-injected mice.

Additionally, tissues were analyzed by fluorescence microscopy, and immune populations in the lung, liver, spleen, and bone marrow that had taken up DiD-labeled exosomes were assessed using flow cytometry.

### Fluorescence microscopy

Harvested tissues were embedded in PELCO® Cryo-Embedding Compound (Ted Pella Inc, cat. #27300), snap frozen, and stored at −80 °C. Embedded frozen tissues were cut into 7-μm thick sections. Sectioned tissues were mounted immediately onto histological slides using ProLong™ Gold Antifade Mountant with DAPI solution (Life Technologies, cat. # P36935). DiD fluorescence emission was detected by either a Zeiss 780-NLO confocal microscope or an Aperio ScanScope FL eSlide capture device. Confocal images were analyzed using the Zen Blue software (ZEISS, v1.1.2.0).

### Flow cytometry

Flow cytometry was carried out on single-cell suspensions of whole lung, spleen, and liver tissues. A standard protocol was used to prepare single-cell suspensions: (i) lungs and liver were minced and then digested with 0.2 mg/ml collagenase type IV (Worthington Biochemical Corp, cat. # LS004189) for 20 and 30 min, respectively, at 37^ o^C; (ii) spleen, and digested lungs and liver tissues were passed through a 70-µm cell strainer to obtain single-cell suspensions, and hepatocytes removed from the latter by Percoll gradient. All cell preparations were treated with ammonium chloride red cell lysis buffer, and re-filtered. Samples were stained with the appropriate antibodies, together with Fc receptor blocking using anti-CD16/32 (1:100 dilution; BD Biosciences, cat. #553142) in PBS containing 2% FBS. Zombie Yellow (1:300 dilution; BioLegend, cat. #423104) was used as a viability dye. Exosome uptake was assessed by detection of DiD-positive cells. Flow-cytometric acquisition was completed using a LSRFortessa™ (BD Biosciences) and the BD FACSDiva™ software (BD Biosciences, v8), and analysis was performed using the FlowJo software (Tree Star, v10.7.1).

### Pre-metastatic niche formation and experimental metastasis

To initiate pre-metastatic niche formation, C57Bl/6 mice were injected intravenously with PBS, 100 pg of mouse recombinant CCL2, or 10^11^ particles, every 48 h, three times. The amount of recombinant CCL2 to be injected per mouse was calculated as an excess based on the average concentration of CCL2 that is found to be bound to exosomes after incubation with either TIF or recombinant CCL2. After exosome injection, lungs, spleen, and liver were harvested, and immune cell composition was assessed using flow cytometry. Alternatively, after exosome conditioning, mice were injected with 10^5^ EO771 cells via the tail vein (experimental metastasis model) and metastatic burden in the lungs was assessed 21 days later. Images of H&E stained lung sections were captured using an Aperio AT turbo brightfield slide scanner, and analyzed by the Image Scope software (Aperio, v12.4.0.5043).

### Exosome conjugation to cytokines

Exosomes (500 µg/ml) were incubated with increasing concentrations of either recombinant CCL2 (human: BioLegend, cat. #571406; mouse: R&D Systems, cat. #479JE-CF) or recombinant IL-6 (human: R&D Systems, cat. #206IL; mouse: Novus Biological, cat. #52156) cytokines in a final volume of 50 µl for 2 h at 37 °C under agitation. To remove free unbound cytokines, exosomes were re-purified using qEV columns (Izon Science Ltd), followed by sample concentration in Amicon Ultra-4 10-kDa nominal molecular weight centrifugal filter units (Merck Millipore) to a final volume of 200 µl. Cytokine binding was then assessed by ELISA. Absorbances were detected using a BioTeK PowerWave HT microplate spectrophotometer and the Gen5™ microplate reader software (BioTek, v2.09).

CCL2-conjugated exosome samples were also submitted to sonication (10 pulses of 5 s at 45% amplitude; 1-min intervals on ice) or treatment with 10 µg/ml of Proteinase K (Sigma-Aldrich, cat. #P2308) for 10 min at 37 °C before CCL2 detection by ELISA. Heat-inactivation of Proteinase K was performed by incubation at 90^ o^C for 5 min.

### ELISA assays

Exosome preparations were assessed by ELISA according to the manufacturer’s instructions. The following ELISA kits were used: mouse CCL2 DuoSet (R&D Systems, cat. #DY479); mouse IL-6 DuoSet (R&D Systems, cat. #DY40605); human CCL2 DuoSet (R&D Systems, cat. #DY279); human IL-6 DuoSet (R&D Systems, cat. #DY20605); human CD44 DuoSet (R&D Systems, cat. #DY704505); human glypican-1 DuoSet (R&D Systems, cat. #DY451905); human syndecan-1 ELISA Set (abcam, cat. #ab47352); human HSPG ELISA Kit (Perlecan) (abcam, cat. #ab274393); human versican ELISA Kit (Novus Biologicals, cat. #NBP275353). Absorbances were detected using a BioTeK PowerWave HT microplate spectrophotometer and the Gen5™ microplate reader software (BioTek, v2.09).

### Treatment of exosomes with heparinase III and/or chondroitinase ABC

Exosomes (500 µg/ml) were incubated with 50 U/ml Heparinase III (Sigma-Aldrich, cat. #H8991) and Chondroitinase ABC (Sigma-Aldrich, cat. #C3667), 1:1 mixture, in a final volume of 200 µl for 90 min at 37 °C under agitation. Control exosomes were incubated with PBS alone. Samples were centrifuged at 100,000 x *g* for 90 min at 4 °C. Exosome pellets were resuspended in PBS for further incubation with cytokines.

### SERS characterization and PCA classification

A cover glass substrate was cleaned by immersing in a piranha solution (H_2_SO_4_: H_2_O_2_ = 3:1) over 30 min to remove organic impurities from the surface. The SERS substrate was prepared by dropping concentrated 100 nm gold nanoparticle colloid onto the cover glass and drying at room temperature. For SERS characterization of the exosomes, 100 μl of the exosome sample (about 10^12^ particles/ml) were dropped onto the substrate and thoroughly dried. We measured the SERS spectra with an inverted microscope (Axio Observer D1, Zeiss) and a spectrometer (PIXIS400 and SP2300, Princeton Instruments). A 785-nm wavelength laser was irradiated to the SERS substrate and the reflected spectral signal was detected through a 50x objective lens (NA = 0.70). The laser power was 5 mW and the acquisition time was 10 s. All spectral data were preprocessed for denoising, baseline correction, and normalization. The PCA was performed using the built-in function of MATLAB 2018. Three hundred Raman signal data per sample were used for the classification.

### Co-immunoprecipitation assay

Exosomes (500 μg/ml) were sonicated (10 pulses of 5 s at 45% amplitude; 1-min intervals on ice) in binding buffer (50 mM Hepes, 150 mM NaCl, 20 mM β-glycerophosphate, 1% NP-40, 0.5% Triton X-100, 1 mM EDTA, 1 mM EGTA, 5% glycerol, pH 7.5), and then incubated with recombinant CCL2 (10 μg/ml) for 1 h 30 min at 37 °C under agitation. Meanwhile, Protein A/G agarose beads (Protein A/G PLUS-Agarose Immunoprecipitation Reagent; Santa Cruz Biotechnology, cat. #2003) were washed according to the manufacturers’ instructions and incubated with 1 μg of anti-CCL2 antibody (BioLegend, cat. #502602) for 1 h at 4 °C. After that, exosome samples were incubated with beads plus antibody overnight at 4 °C under gentle agitation. Beads conjugated with immune-captured samples were washed five times with binding buffer. To elute proteins from the beads, samples were incubated with 20 μl of Laemli buffer at 95 °C for 10 min. Protein content in the supernatant was finally analyzed by either western blotting or ELISA.

### Statistical analyses

Prism software (GraphPad, v8) was used for statistical analysis. Data are presented as the mean ± SEM of results obtained from at least three independent experiments. Mean differences were compared using two-tailed Mann–Whitney *U*-tests, with *p* < 0.05 considered to be statistically significant.

### Reporting summary

Further information on research design is available in the Nature Research Reporting Summary linked to this article.

## Supplementary information

Supplementary Information

Reporting Summary

## Data Availability

The data supporting this study are available in the Article, Supplementary Information, Source Data, and from the authors upon reasonable request. Source data are provided with this paper.

## Source data

Source Data
