# Supplementary material for: Tumor microenvironmental cytokines bound to cancer exosomes determine uptake by cytokine receptor-expressing cells and biodistribution
Source: Nat Commun. 2021 Jun 10;12:3543. doi: 10.1038/s41467-021-23946-8 (PMC8192925; doi:10.1038/s41467-021-23946-8)
Supplement: Supplementary file 1 — Supplementary Information [file 41467_2021_23946_MOESM1_ESM.pdf]

Supplementary Information

Tumor microenvironmental cytokines bound to cancer exosomes determine uptake by cytokine receptor-expressing cells and biodistribution

Luize G Lima[1,a], Sunyoung Ham[1,2,a], Hyunku Shin[3], Edna P Z Chai[1,4], Erica S H Lek[1,5], Richard J Lobb[1,b], Alexandra F Müller[1], Suresh Mathivanan[6], Belinda Yeo[7], Yeonho Choi[3,8,9], Belinda S Parker[10,11] and Andreas Möller[1,2,4]*

Supplementary material includes:

- Supplementary Figures and Legends 1-8
- Supplementary Table 1
- Supplementary Table 2

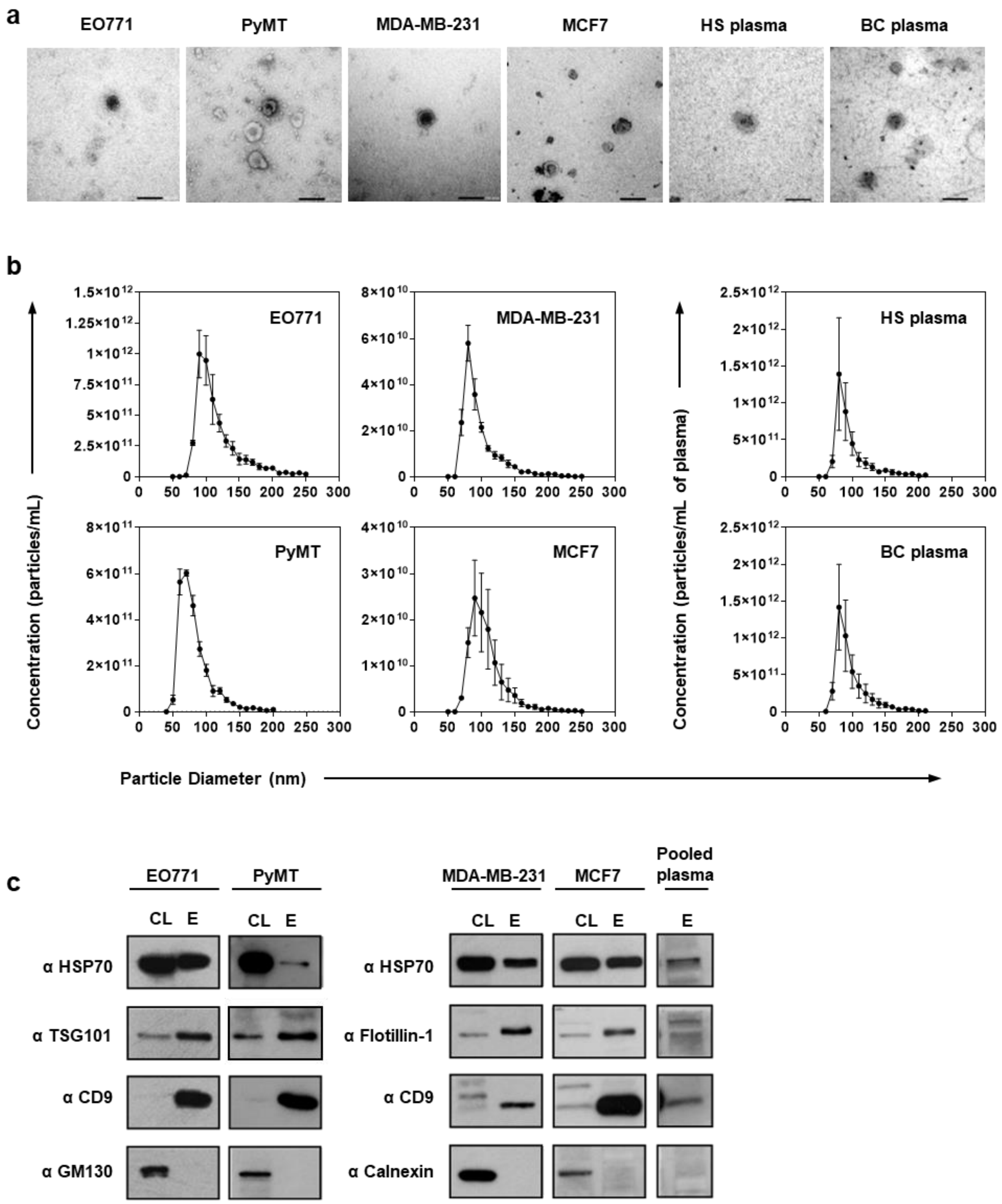

Supplementary Fig. 1 Characterization of purified exosomes. a Morphology of exosomes isolated from the conditioned media of murine (EO771, PyMT) and human (MDA-MB-231, MCF7) breast cancer cells, or purified from human plasma samples, as assessed by transmission electron microscopy. HS: healthy subjects; BC: breast cancer patients. Size bar = 200 nm. Representative images of at least 10 different fields obtained from one exosome

preparation are shown. **b** Size distribution and enumeration of particles as assessed by TRPS (EO771, MDA-MB-231, MCF7, and HS plasma, n=3; PyMT, n=4; BC plasma, n=6; all biologically independent exosome preparations). Each data point represents mean ± SEM of the respective size bin. **c** Expression of exosomal (CD9, 25 kDa; TSG101, 47 kDa; Flotillin-1, 48kDa; HSP70, 70 kDa) and cell (endoplasmic reticulum protein Calnexin, 67kDa; cis-Golgi protein GM130, 112 kDa) markers in cell lysates (CL) and exosomes (E) isolated from cell conditioned media or pooled HS/BC plasma as indicated. Similar results were obtained in at least 3 biologically independent exosome preparations. Source data are provided as a Source Data file.

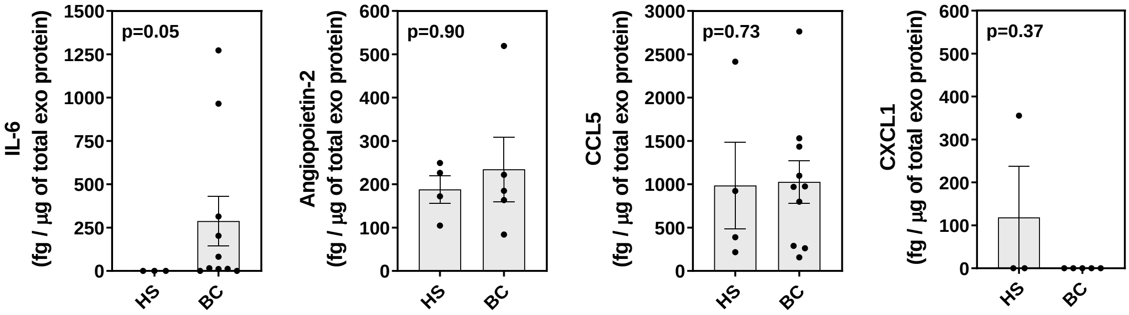

Supplementary Fig. 2 Quantification of IL-6, Angiopoietin-2, CCL5 and CXCL1. The abundance of IL-6 (n=3 HS and n=10 BC biologically independent exosome preparations), Angiopoietin-2 (n=4 HS and n=5 BC biologically independent exosome preparations), CCL5 (n=4 HS and n=10 BC biologically independent exosome preparations) and CXCL5 (n=3 HS and n=5 BC biologically independent exosome preparations) was quantified by ELISA in exosomes purified from plasma of healthy subjects (HS) or BC patients. Data are presented as mean ± SEM. p values are shown for each cytokine as analyzed by two-tailed Mann–Whitney U test.

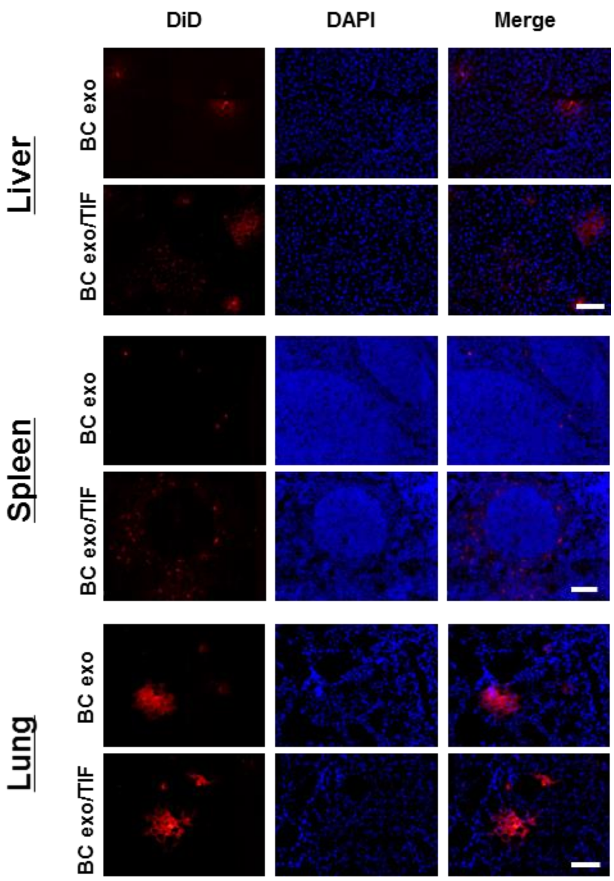

Supplementary Fig. 3 Increased retention of TIF-conjugated exosomes in different organs. DiD-labelled EO771 exosomes previously incubated (BC exo/TIF, n=7 animals) or not (BC exo, n=8 animals) with TIF were injected into C57Bl/6 WT mice, and biodistribution assessed 24 h after injection. Fluorescence microscopy-based evaluation of liver, spleen and lung sections shows increased DiD signal intensity in tissues of mice that received TIF-conjugated exosomes. Size bar = 100 µm.

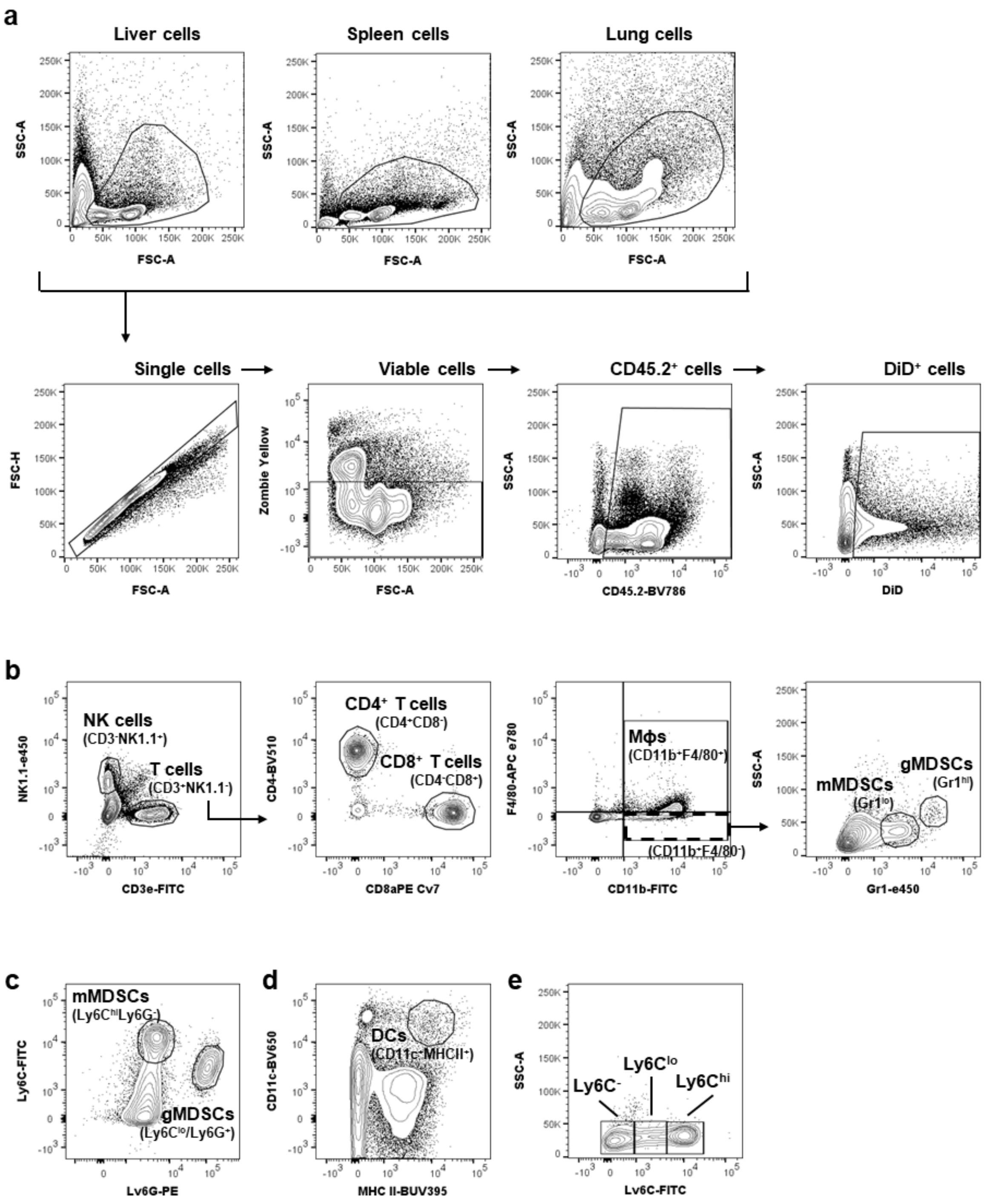

Supplementary Fig. 4 Gating strategy for flow cytometry analysis. a Analyses of single-cell suspensions of liver, spleen and lung tissues were performed on live single cells, chosen according to their FSC and SSC parameters. Zombie Yellow was used as a viability dye. Leucocytes were selected as CD45.2$^+$ cells, and frequency of DiD$^+$ population was assessed within the leucocyte population. **b** Distinct CD45.2$^+$ immune cell subsets were selected as: CD3$^-$NK1.1$^+$, for NK cells; CD3$^+$NK1.1$^-$ for T cells, which were further gated as CD4$^+$ or CD8$^+$

T cells; $CD11b^+F4/80^+$, for macrophages (Mϕ); $CD11b^+Gr1^{lo}$, for mMDSCs; and $CD11b^+Gr1^{hi}$, for gMDSCs. Frequency of DiD^+ population was also assessed within each $CD45.2^+$ immune cell subset. **c** Alternatively, MDSCs were selected from $CD11b^+F4/80^-$ cells as: $CD11b^+Ly6C^{hi}Ly6G^-$, for mMDSCs; and $CD11b^+/Ly6C^{lo}/Ly6G^+$, for gMDSCs. **d** Dendritic cells (DCs) were eventually selected from $CD45.2^+$ cells as $CD11c^+MHCII^+$. **e** $Ly6C^-$, $Ly6C^{lo}$ and $Ly6C^{hi}$ populations were eventually selected within macrophages (Mϕ). Representative images are shown.

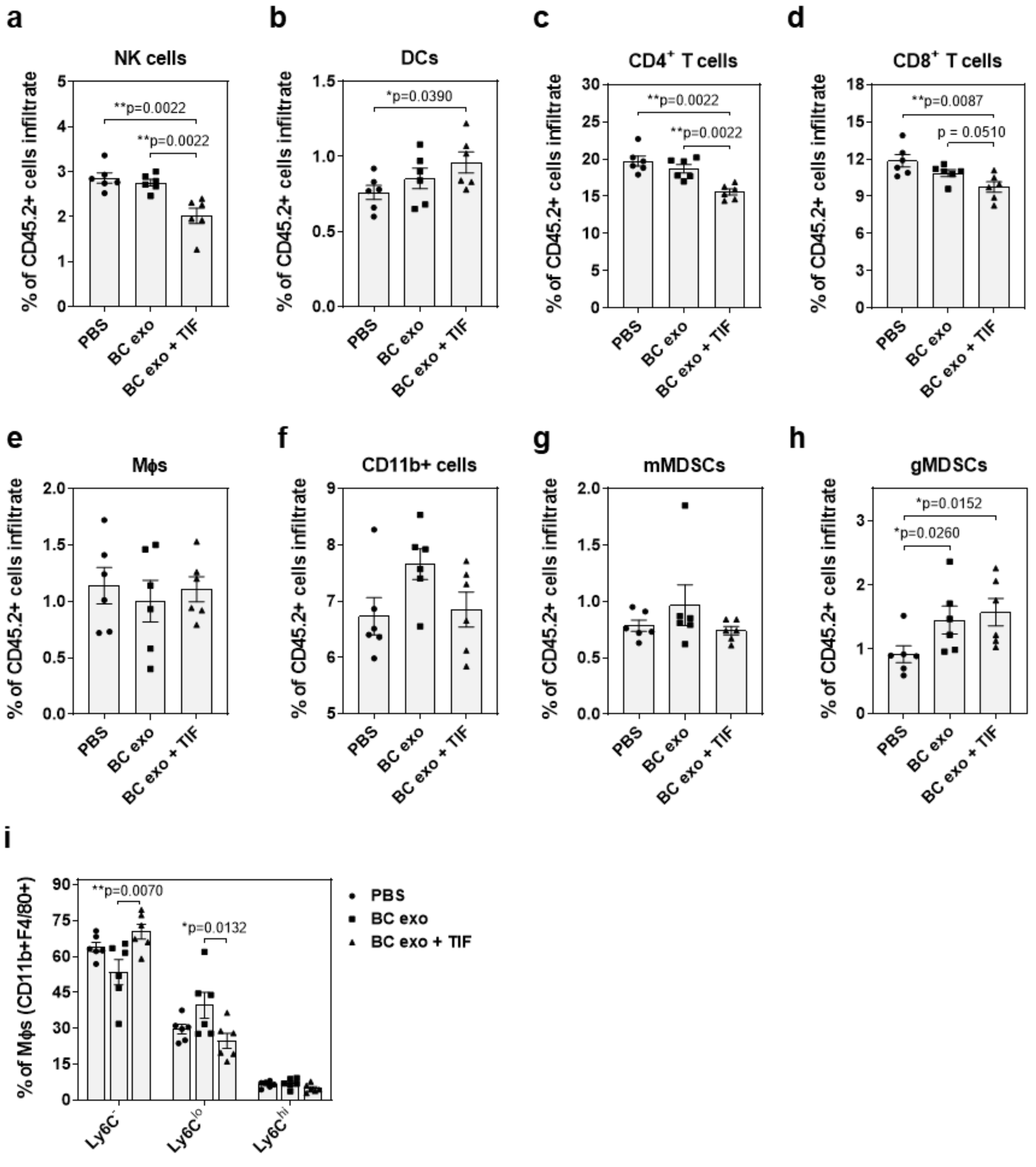

Supplementary Fig. 5 Effect of TIF-conjugated exosomes on the frequency of immune cell subsets in the spleen. C57Bl/6 WT mice were injected with EO771 exosomes previously incubated (BC exo/TIF) or not (BC exo) with TIF, every 48 h, 3 times. PBS only-injected mice served as negative controls (n=6 animals/group). **a-h** Frequency of distinct CD45.2$^+$ immune cell subsets: (**a**) NK cells (CD3$^-$NK1.1$^+$), (**b**) dendritic cells (DCs; CD11c$^+$MHCII$^+$), (**c**) CD4$^+$ T cells (CD3$^+$CD4$^+$), (**d**) CD8$^+$ T cells (CD3$^+$CD8$^+$), (**e**) macrophages (Mϕ; CD11b$^+$F4/80$^+$), (**f**) myeloid cells (CD11b$^+$), (**g**) mMDSCs (CD11b$^+$Ly6C^{hi}Ly6G$^-$), and (**h**) gMDSCs (CD11b$^+$/Ly6C^{lo}/Ly6G$^+$) as assessed by flow cytometry. **i** Frequency of Ly6C$^-$, Ly6C^{lo} and Ly6C^{hi} cells within macrophage population. Data are presented as mean ± SEM. *, $p < 0.05$, and **, $p < 0.01$ as analyzed by two-tailed Mann–Whitney U test.

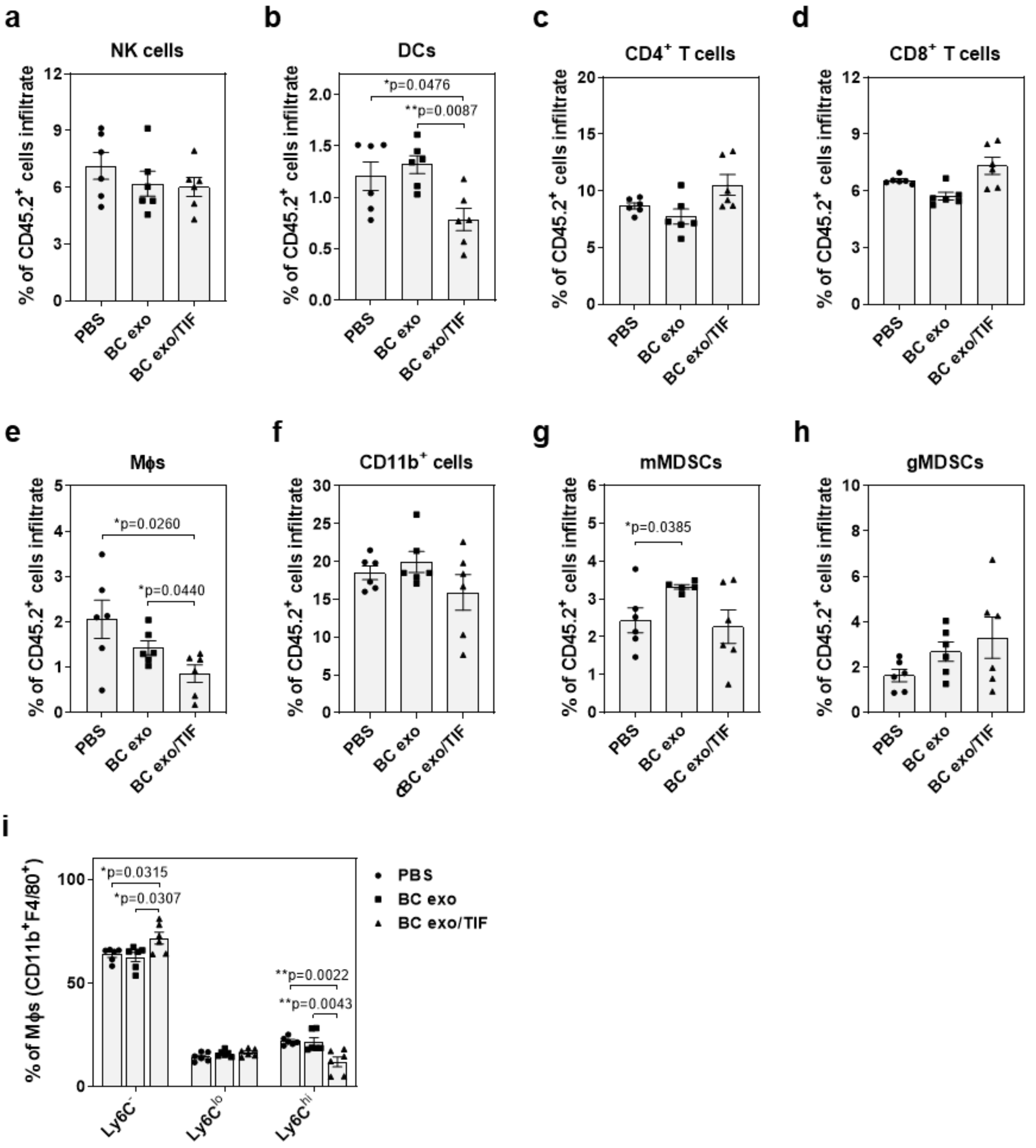

Supplementary Fig. 6 Effect of TIF-conjugated exosomes on the frequency of immune cell subsets in the liver. C57Bl/6 WT mice were injected with EO771 exosomes previously incubated (BC exo/TIF) or not (BC exo) with TIF, every 48 h, 3 times. PBS only-injected mice served as negative controls (n=6 animals/group). **a-h** Frequency of distinct CD45.2$^+$ immune cell subsets: (a) NK cells (CD3$^-$NK1.1$^+$), (**b**) dendritic cells (DCs; CD11c$^+$MHCII$^+$), (**c**) CD4$^+$ T cells (CD3$^+$CD4$^+$), (**d**) CD8$^+$ T cells (CD3$^+$CD8$^+$), (**e**) macrophages (Mϕ; CD11b$^+$F4/80$^+$), (**f**) myeloid cells (CD11b$^+$), (**g**) mMDSCs (CD11b$^+$Ly6C^{hi}Ly6G$^-$), and (**h**) gMDSCs (CD11b$^+$/Ly6C^{lo}/Ly6G$^+$) as assessed by flow cytometry. **i** Frequency of Ly6C$^-$, Ly6C^{lo} and Ly6C^{hi} cells within macrophage population. Data are presented as mean ± SEM. *, $p < 0.05$, and **, $p < 0.01$ as analyzed by two-tailed Mann–Whitney U test.

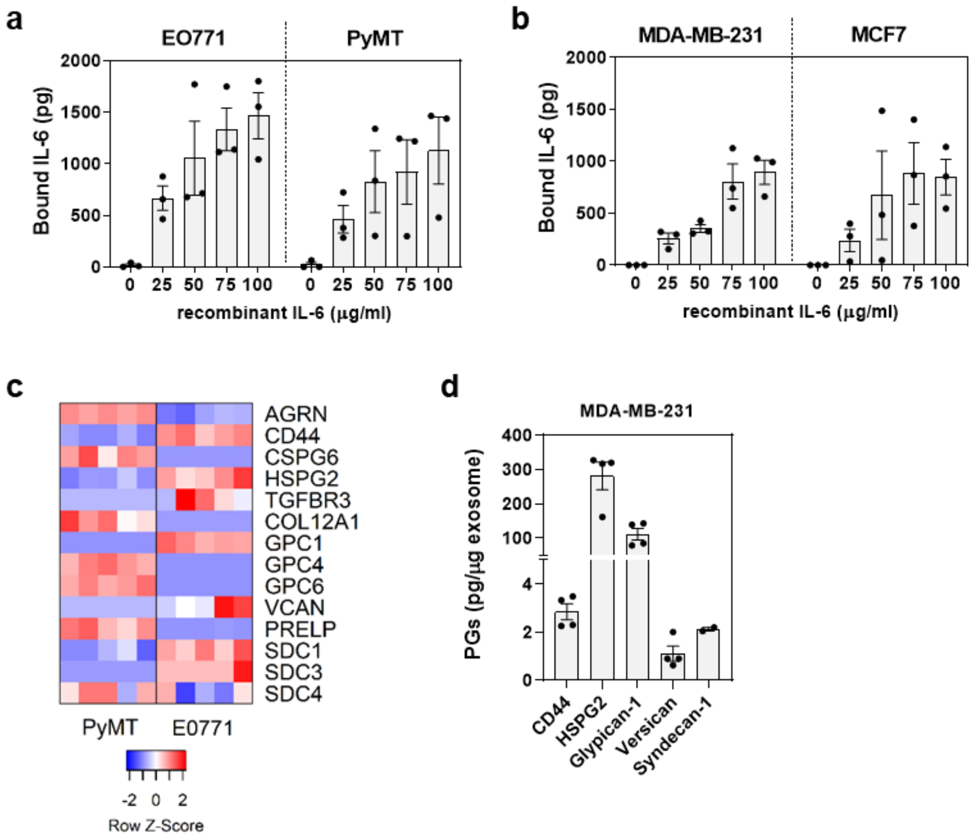

Supplementary Fig. 7 IL-6 binds to the surface of murine and human breast cancer cell-derived exosomes, which display various proteoglycans. a, b Concentration-dependent binding of IL-6 to murine (**a**, EO771 and PyMT) and human (**b**, MDA-MB-231 and MCF7) breast cancer cell culture-derived exosomes (n=3 independent experiments/group). **c** Heatmap of mass spectrometry data (from (11)) analysing abundances of different proteoglycans (AGRN, CD44, CSPG6, HSPG2, TGFBR3, COL12A1, GPC1, GPC4, GPC6, VCAN, PRELP, SDC1, SDC3, SDC4) in exosomes derived from PyMT and EO771 cells. **d** Quantitative analysis of the presence of different proteoglycans (CD44; HSPG2; Glypican-1, GPC1; Versican, VCAN; Syndecan-1 SDC1) in MDA-MB-231 exosomes by ELISA (n=4 biologically independent exosome preparations, except for Syndecan-1 (n=2)). Data are presented as mean ± SEM.

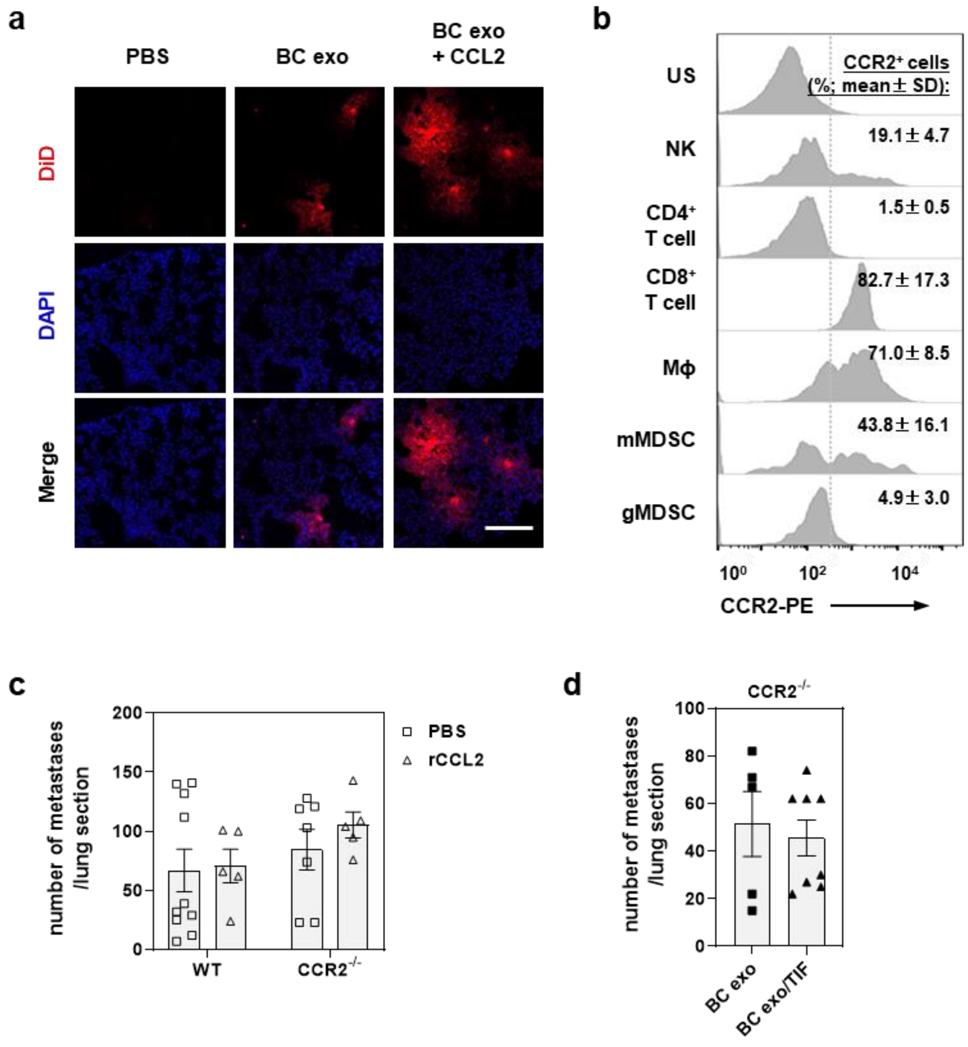

Supplementary Fig. 8 CCL2-conjugated exosomes accumulate in the lungs of WT mice. **a** DiD-labelled EO771 exosomes previously incubated (BC exo + CCL2, n=3 animals) or not (BC exo, n=4 animals) with CCL2 were injected into C57Bl/6 WT mice. Fluorescence microscopy-based evaluation of lung shows increased DiD signal intensity in tissues of mice that received CCL2-conjugated exosomes. Size bar = 100 µm. **b** Flow-cytometric assessment of CCR2 expression in distinct CD45.2$^+$ immune cell subsets within the lung (n=4 biologically independent samples/group): NK cells (CD3$^-$NK1.1$^+$); CD4$^+$ T cells (CD3$^+$CD4$^+$); CD8$^+$ T cells (CD3$^+$CD8$^+$); macrophages (Mϕ; CD11b$^+$F4/80$^+$); mMDSCs (CD11b$^+$Gr1^{lo}); and gMDSCs (CD11b$^+$/Gr1^{hi}). **c** Mice were pre-conditioned by repeated injections of PBS (n=10 and n=7 animals in WT and CCR2$^{-/-}$ groups, respectively) or recombinant CCL2 (n=7 and n=5 animals in WT and CCR2$^{-/-}$ groups, respectively), and then received a single i.v. injection of EO771 cells. Metastatic burden in the lung was assessed after 21 days. **d** Mice pre-conditioned with cell culture-derived EO771 exosomes previously incubated (BC exo/TIF, n=8 animals) or

not (BC exo, n=5 animals) with TIF received a single i.v. injection of EO771 cells, and metastatic burden in the lung was assessed. Numbers of metastatic foci per tissue section are shown in both (**c**) and (**d**) (each data point represents a different mouse; one section was examined per mouse; all 5 lobes were analyzed and combined to determine total number of metastases per section). Data are presented as mean ± SEM.

Supplementary Table 1. Characteristics of patients and healthy subjects.

	**BC patients* (n = 12)**	**Healthy subjects (n = 6)**
**Age at sample collection**		
Median (range)	50 (37-69)	67 (35-71)
**BC subtype**		
Luminal	3 (25%)	
$HER2^{+}$	7 (58%)	
TNBC	2 (17%)	
**IHC status**		
ER^{+} / PR^{+} / HER^{-}	3 (25%)	
ER^{+} / $PR^{+/-}$ / HER^{+}	4 (33%)	
ER^{-} / PR^{-} / HER^{+}	3 (25%)	
ER^{-} / PR^{-} / HER^{-}	2 (17%)	

**All patients presented with stage IV disease at time of sample collection.*

Supplementary Table 2. List of cytokine receptors identified in EO771 and PyMT cell-derived exosomes by mass spectrometry data (11).

Protein name	Gene name	No. of distinct peptides	PSMs
Platelet-derived growth factor receptor alpha	PDGFRA	24	166
Fibroblast growth factor receptor 1	FGFR1	19	122
Platelet-derived growth factor receptor beta	PDGFRB	18	109
Epidermal growth factor receptor	EGFR	25	88
Interleukin-6 receptor subunit beta	IL6RB	16	88
Tumor necrosis factor receptor superfamily member 10B	DR5	8	64
TGF-beta receptor type-1	TGFBR1	6	42
Hepatocyte growth factor receptor	MET	13	33
Interleukin-17 receptor A	IL17RA	7	30
Tumor necrosis factor receptor superfamily member 1A	TNFR1	5	21
TGF-beta receptor type-2	TGFBR2	7	13
Transforming growth factor beta receptor type-3	TGFBR3	6	13
