# Supplementary material for: Tumor microenvironmental cytokines bound to cancer exosomes determine uptake by cytokine receptor-expressing cells and biodistribution
Source: Nat Commun. 2021 Jun 10;12:3543. doi: 10.1038/s41467-021-23946-8 (PMC8192925; doi:10.1038/s41467-021-23946-8)
Supplement: Supplementary file 2 — Reporting Summary [file 41467_2021_23946_MOESM2_ESM.pdf]

nature research

Corresponding author(s): Andreas Möller

Last updated by author(s): 19/05/2021

Reporting Summary

Nature Research wishes to improve the reproducibility of the work that we publish. This form provides structure for consistency and transparency in reporting. For further information on Nature Research policies, see our Editorial Policies and the Editorial Policy Checklist.

Statistics

For all statistical analyses, confirm that the following items are present in the figure legend, table legend, main text, or Methods section.

n/a	Confirmed	
☐	☒	The exact sample size (*n*) for each experimental group/condition, given as a discrete number and unit of measurement
☐	☒	A statement on whether measurements were taken from distinct samples or whether the same sample was measured repeatedly
☐	☒	The statistical test(s) used AND whether they are one- or two-sided<br>*Only common tests should be described solely by name; describe more complex techniques in the Methods section.*
☒	☐	A description of all covariates tested
☒	☐	A description of any assumptions or corrections, such as tests of normality and adjustment for multiple comparisons
☐	☒	A full description of the statistical parameters including central tendency (e.g. means) or other basic estimates (e.g. regression coefficient) AND variation (e.g. standard deviation) or associated estimates of uncertainty (e.g. confidence intervals)
☐	☒	For null hypothesis testing, the test statistic (e.g. *F*, *t*, *r*) with confidence intervals, effect sizes, degrees of freedom and *P* value noted<br>*Give P values as exact values whenever suitable.*
☒	☐	For Bayesian analysis, information on the choice of priors and Markov chain Monte Carlo settings
☒	☐	For hierarchical and complex designs, identification of the appropriate level for tests and full reporting of outcomes
☒	☐	Estimates of effect sizes (e.g. Cohen's *d*, Pearson's *r*), indicating how they were calculated

Our web collection on statistics for biologists contains articles on many of the points above.

Software and code

Policy information about availability of computer code

Data collection	Biotek Gen5™ microplate reader software v2.09 (Bradford, BCA and ELISA assays); BD FACSDiva™ software v8 (acquisition of flow cytometric data); PerkinElmer Living Image software v4.4 (acquisition of fluorescence images of harvested tissues).
Data analysis	ImageJ software v1.51w (analysis of cytokine array data); PerkinElmer Living Image software v4.4 (analysis of fluorescence images obtained by IVIS Spectrum system); Tree Star FlowJo software v10.7.1 (analysis of flow cytometric data); BD FCAP Array™ software v3 (analysis of CBA data); ZEISS Zen Blue software v1.1.2.0 (analysis of fluorescence images obtained by confocal microscopy); Aperio Image Scope software v12.4.0.5043 (analysis of H&E stained tissue sections); MATLAB 2018 software (analysis of SERS data); GraphPad Prism software v8 (data plotting and statistical analysis).

For manuscripts utilizing custom algorithms or software that are central to the research but not yet described in published literature, software must be made available to editors and reviewers. We strongly encourage code deposition in a community repository (e.g. GitHub). See the Nature Research guidelines for submitting code & software for further information.

Data

Policy information about availability of data

All manuscripts must include a data availability statement. This statement should provide the following information, where applicable:

- Accession codes, unique identifiers, or web links for publicly available datasets
- A list of figures that have associated raw data
- A description of any restrictions on data availability

The data supporting the findings of this study are available from the corresponding author upon reasonable request.

Field-specific reporting

Please select the one below that is the best fit for your research. If you are not sure, read the appropriate sections before making your selection.

☒ Life sciences ☐ Behavioural & social sciences ☐ Ecological, evolutionary & environmental sciences

For a reference copy of the document with all sections, see nature.com/documents/nr-reporting-summary-flat.pdf

Life sciences study design

All studies must disclose on these points even when the disclosure is negative.

Sample size	No statistical methods were used to calculate sample size for in vitro experiments. At least 3 biological replicates were examined over independent experiments for every treatment tested (as stated in figure legends), with reproducible results. In some cases, sample number was increased to improve statistical power. In vivo assays included 5-9 mice per treatment group. Sample size was chosen based on previous experience of our group (Wen et al., DOI: 10.1158/0008-5472.CAN-16-0868), as well as on other publications of similar field/nature (Hoshino et al., DOI: 10.1038/nature15756; Olmeda et al., DOI: 10.1038/nature22977), and in order to achieve a statistical power of approximately 80% (alpha=0.05).
Data exclusions	No data were excluded.
Replication	Experiments were repeated at least 3 times with distinct samples, and were successful.<br>In vitro assays such as Bradford/BCA, BD™ CBA Flex Set and ELISA were performed with 2 or 3 technical replicates.
Randomization	Mice were randomly assigned to experimental groups.<br>Exosome preparations were randomly assigned to the different treatment conditions tested throughout the study.
Blinding	No blinding was performed, since most of our assays involved empirical measurements and not subjective analysis.<br>All experimental analyses were consistently conducted by the same person, and independently verified by another investigator for validation.

Reporting for specific materials, systems and methods

We require information from authors about some types of materials, experimental systems and methods used in many studies. Here, indicate whether each material, system or method listed is relevant to your study. If you are not sure if a list item applies to your research, read the appropriate section before selecting a response.

Materials & experimental systems

n/a	Involved in the study	
☐	☒	Antibodies
☐	☒	Eukaryotic cell lines
☒	☐	Palaeontology and archaeology
☐	☒	Animals and other organisms
☐	☒	Human research participants
☒	☐	Clinical data
☒	☐	Dual use research of concern

Methods

n/a	Involved in the study	
☒	☐	ChIP-seq
☐	☒	Flow cytometry
☒	☐	MRI-based neuroimaging

Antibodies

Antibodies used	The following antibodies were used for western blotting: anti-flotillin-1 (BD Biosciences, cat. #610821, clone 18/Flotillin-1), anti-HSP70 (BD Biosciences, cat. #610608, clone 7/Hsp70), anti-TSG101 (Santa Cruz Biotechnology, cat. #6037, clone M-19), anti-CD9 (Abcam, cat. #92726, clone:EPR2949), anti-GM130 (Abcam, cat. #52649, clone EP892Y), anti-calnexin (Cell Signalling Technology, cat. #2679S, clone C5C9), anti-syndecan-1 (Abcam, cat. #128936, clone EPR6454), anti-versican (Abcam, cat. #177480, clone EPR12277), anti-CCL2 (BioLegend, cat. #502602, clone 5D3-F7), anti-CD44 (eBioscience, cat. #11-0441-81, clone IM7), anti-mouse HRP-conjugated (Pierce, cat. #1858413), anti-rabbit HRP-conjugated (Pierce, cat. #1858415), anti-goat HRP-conjugated (Sigma-Aldrich, cat. #A5420), and anti-rat HRP-conjugated (XXXXX).<br><br>The following antibodies were used for flow cytometry: anti-CCR2 PE (R&D Systems, cat. #FAB5538P, clone 475301), anti-CCR4 APC (BioLegend, cat. #131211, clone 2G12), anti-CD45.2 BV786 (BD Biosciences, cat. #563686, clone 104), anti-CD3e FITC (eBioscience, cat. #11-003181, clone 145-2C11), anti-NK1.1 e450 (eBioscience, cat. #48-5941-80, clone PK136), anti-CD8a PE (BD Biosciences, cat. #553033, clone 53-6.7), anti-CD8a PE-Cy7 (eBioscience, cat. #25-0081-81, clone 53-6.7), anti-CD4 BV510 (BD Biosciences, cat. #563106, clone RM4-5), anti-CD11c BV650 (BD Biosciences, cat. #564079, clone HL3), anti-Gr1 e450 (eBioscience, cat. #48-5931-80, clone RB6-8C5), anti-CD11b FITC (eBioscience, cat. #11-0112-81 clone M1/70), anti-CD11b PE-Cy7 (eBioscience, cat. #25-0112-81, clone M1/70), anti-F4/80 APC-e780 (eBioscience, cat. #47-4801-80, clone BM8), anti-Ly6C FITC (BD Biosciences, cat. #553104, clone AL-21), anti-Ly6G PE (BD Biosciences, cat. #220461, clone 1A8), anti-MHCII BUV395 (BD Biosciences, cat. #743876, clone 2G9).

April 2020

Validation	The following antibodies were validated in Lobb et al. (DOI: 10.3402/jev.v4.27031): anti-flotillin-1, anti-HSP70, anti-TSG101, anti-calnexin, anti-mouse HRP-conjugated, anti-goat HRP-conjugated, and anti-rabbit HRP-conjugated.<br><br>The following antibodies were validated in Wen et al. (DOI: 10.1158/0008-5472.CAN-16-0868): anti-CD9, anti-GM130, anti-CD45.2 BV786, anti-CD3e FITC, anti-NK1.1 e450, anti-CD8a PE, anti-CD8a PE-Cy7, anti-CD4 BV510, anti-CD11c BV650, anti-Gr1 e450, anti-CD11b FITC, anti-CD11b PE-Cy7, anti-F4/80 APC-e780, anti-Ly6C FITC, anti-Ly6G PE and anti-MHCII BUV395.<br><br>Anti-syndecan-1 was validated in Wang et al. (DOI: 10.3892/or.2018.6271).<br><br>Anti-versican antibody was validated in Evanko et al. (DOI: 10.1016/bs.mcb.2017.08.015).<br><br>Anti-CCL2 antibody was validated in Hirsch et al. (DOI: 10.1128/JVI.73.1.404-410.1999).<br><br>Anti-CCR2 PE mouse and anti-CCR4 APC antibodies were validated in Goudot et al. (DOI: 10.1016/j.immuni.2017.08.016.) and Kishore et al. (DOI: 10.1016/j.immuni.2017.10.017.).<br><br>Anti-CD44 antibody was validated in Yang et al. (DOI: 10.1074/jbc.M112.349209Anti-rat-HRP conjugated was validated in Ham et al. (DOI: 10.3389/fimmu.2018.00871).

Eukaryotic cell lines

Policy information about cell lines

Cell line source(s)	EO771 cells were a kind gift from Robin Anderson, Peter MacCallum Cancer Centre, Melbourne (the EO771 cell line was derived from a spontaneous mammary tumour in a C57BL/6 mouse in 1951 (Casey et al., DOI: 10.3181/00379727-77-18779), and stored in liquid nitrogen vapour phase); PyMT cells were generated by our group as in Wong et al. (DOI: 10.1158/0008-5472.CAN-11-3310); MDA-MB-231 and MCF-7 cell lines were both obtained from ATCC.
Authentication	Authentication of both human MDA-MB-231 and MCF-7 cell lines was verified using in-house STR profiling. EO771 and PyMT murine cell lines were not authenticated.
Mycoplasma contamination	All cell lines were routinely tested negative for mycoplasma contamination.
Commonly misidentified lines<br>(See ICLAC register)	No commonly misidentified cell lines were used in the study.

Animals and other organisms

Policy information about studies involving animals; ARRIVE guidelines recommended for reporting animal research

Laboratory animals	Female C57Bl/6 wild-type mice were used at 8-10 weeks of age and purchased from the Walter and Eliza Hall Institute (Melbourne, Australia). C57Bl/6 CCR2-/- mice were bred and maintained at the QIMR Berghofer Medical Research Institute.<br>Animals were housed in passive air flow EVC (environmentally ventilated cage) mice system. These mouse cages are housed in an environmentally controlled room that is maintained at 20oC-21oC, 60% relative humidity and a 12 hour light cycle (8am to 8pm) with no external or natural light sources. All exhaust air from the mouse racks is vented external to the building.
Wild animals	No wild animals were used in the study.
Field-collected samples	No field collected samples were used in the study.
Ethics oversight	All animal procedures were conducted in accordance with Australian National Health and Medical Research regulations on the use and care of experimental animals, and approved by the QIMR Berghofer Medical Research Institute Animal Ethics Committee (A12617M, P1499).

Note that full information on the approval of the study protocol must also be provided in the manuscript.

Human research participants

Policy information about studies involving human research participants

Population characteristics	Plasma samples were prepared from blood collected from healthy female, age-matched subjects, or breast cancer patients. Population characteristics are described in Supplementary Table 1.
Recruitment	Participants were recruited by involved clinicians. Patients were not selected for treatment, and blood samples were collected during standard clinical procedures. Results of the study were not available for participating clinicians or patients, thereby excluding bias.
Ethics oversight	Ethical clearance for the use of the plasma samples was granted by the QIMR Berghofer Medical Research Institute Human Research Ethics Committee (P1499).

Note that full information on the approval of the study protocol must also be provided in the manuscript.

Flow Cytometry

Plots

Confirm that:

☒ The axis labels state the marker and fluorochrome used (e.g. CD4-FITC).

☒ The axis scales are clearly visible. Include numbers along axes only for bottom left plot of group (a 'group' is an analysis of identical markers).

☒ All plots are contour plots with outliers or pseudocolor plots.

☒ A numerical value for number of cells or percentage (with statistics) is provided.

Methodology

Sample preparation	Flow cytometry was carried out on single-cell suspensions of whole spleen, lung and liver tissues. A standard protocol was used to prepare single-cell suspensions: (i) lungs and liver were minced and then digested with 0.2 mg/ml collagenase type IV (Worthington Biochemical Corp, cat. # LS004189) for 20 and 30 minutes, respectively, at 37oC; (ii) spleen, and digested lungs and liver tissues were passed through a 70-µm cell strainer to obtain single-cell suspensions, and hepatocytes removed from the latter by Percoll gradient. All cell preparations were treated with ammonium chloride red cell lysis buffer, and re-filtered. Samples were stained with the appropriate antibodies, together with Fc receptor blocking using anti-CD16/32 (BD Biosciences, cat. #553142) in PBS containing 2% FBS. Zombie Yellow (BioLegend, cat. #423104) was used as a viability dye.
Instrument	Flow-cytometric acquisition was completed using a LSRFortessa (BD Biosciences).
Software	Analysis was performed using the FlowJo software (Tree Star, v10.7.1).
Cell population abundance	Abundance of viable single CD45+ cells in all the samples assessed was at least 80%.
Gating strategy	As described in Supplementary Figure 4, analyses of single-cell suspensions of spleen, lung and liver tissues were performed on live single cells, chosen according to their FSC and SSC parameters. Zombie Yellow was used as a viability dye. Leucocytes were selected as CD45.2+ cells, and frequency of DiD+ population was assessed within the leucocyte population. Distinct CD45.2+ immune cell subsets were selected as: CD3-NK1.1+, for NK cells; CD3+NK1.1- for T cells, which were further gated as CD4+ or CD8+ T cells; CD11b+F4/80+, for macrophages (Mϕ); CD11b+Gr1lo, for mMDSCs; and CD11b+Gr1hi, for gMDSCs. Frequency of DiD+ population was also assessed within each CD45.2+ immune cell subset. Alternatively, MDSCs were selected from CD11b+F4/80- cells as: CD11b+Ly6ChiLy6G-, for mMDSCs; and CD11b+/Ly6Clo/Ly6G+, for gMDSCs. Dendritic cells (DCs) were eventually selected from CD45.2+ cells as CD11c+MHCII+. Ly6C-, Ly6Clo and Ly6Chi populations were eventually selected within macrophages (Mϕ).

☒ Tick this box to confirm that a figure exemplifying the gating strategy is provided in the Supplementary Information.
